# Cell Surface Proteome of Dental Pulp Stem Cells Identified by Label-Free Mass Spectrometry

**DOI:** 10.1371/journal.pone.0159824

**Published:** 2016-08-04

**Authors:** Christian Niehage, Jana Karbanová, Charlotte Steenblock, Denis Corbeil, Bernard Hoflack

**Affiliations:** Biotechnology Center (BIOTEC), Technische Universität Dresden, Dresden, Germany; Second University of Naples, ITALY

## Abstract

Multipotent mesenchymal stromal cells (MSCs) are promising tools for regenerative medicine. They can be isolated from different sources based on their plastic-adherence property. The identification of reliable cell surface markers thus becomes the Holy Grail for their prospective isolation. Here, we determine the cell surface proteomes of human dental pulp-derived MSCs isolated from single donors after culture expansion in low (2%) or high (10%) serum-containing media. Cell surface proteins were tagged on intact cells using cell impermeable, cleavable sulfo-NHS-SS-biotin, which allows their enrichment by streptavidin pull-down. For the proteomic analyses, we first compared label-free methods to analyze cell surface proteomes i.e. composition, enrichment and proteomic differences, and we developed a new mathematical model to determine cell surface protein enrichment using a combinatorial gene ontology query. Using this workflow, we identified 101 cluster of differentiation (CD) markers and 286 non-CD cell surface proteins. Based on this proteome profiling, we identified 14 cell surface proteins, which varied consistently in abundance when cells were cultured under low or high serum conditions. Collectively, our analytical methods provide a basis for identifying the cell surface proteome of dental pulp stem cells isolated from single donors and its evolution during culture or differentiation. Our data provide a comprehensive cell surface proteome for the precise identification of dental pulp-derived MSC populations and their isolation for potential therapeutic intervention.

## Introduction

Multipotent human mesenchymal stromal cells (MSCs) [1], initially described as colony-forming unit-fibroblasts [2, 3], are non-hematopoietic progenitors present in many tissues. MSCs have the remarkable property of differentiating into a variety of cell types while self-renewing. MSCs are considered as promising candidates for tissue engineering and regenerative medicine [4], because they are also able to migrate to injured tissues and to suppress responses linked with immunity [5] or inflammation [6–8]. Pluripotent embryonic stem cells (ESCs) also exhibit these properties and, beside ethical issues they could as well be considered for therapeutic intervention. However, their use for therapeutic intervention remains limited due to the observation that donor-derived tumors can develop after ESC transplantation [9].

The bone marrow has been considered as a main source of MSCs. However, the collection of bone marrow from patients is an invasive procedure, and other tissue sources may be more suitable for therapeutic intervention. Dental pulp tissues have been investigated as niches of MSCs, and many tooth-derived stem cells have been identified and characterized (for recent reviews see [10–12]). Dental pulp tissues are an easy accessible source of MSCs as extracted/exfoliated teeth represent a waste product of dental procedures. The first tooth-derived stem cells to be isolated and characterized were the dental pulp stem cells (DPSCs) [13]. As MSCs, they have a high proliferative potential and are capable of differentiating into osteoblasts, chondroblasts, neurons, liver cells and β cells of islet of the pancreas [14–16] making it possible to use these stem cells for future regenerative therapies of various diseases.

Generally, isolated MSCs have been cultured in media containing a high content of serum (10%). However, such serum concentration for long-term culture might lead to spontaneous differentiation [17, 18] or malignant transformation [19–21]. We have previously shown that DPSCs can efficiently be expanded in low serum-containing (2%) medium supplemented with epidermal growth factor (EGF) and platelet-derived growth factor BB (PDGF-BB). These DPSCs maintain a stable karyotype and they fully maintain their differentiation capabilities [14, 15].

Cell surface antigens are commonly used as biomarkers to characterize and/or isolate various cell types using antibody-based methods such as fluorescence-activated cell sorting (FACS) or paramagnetic selection. However, alternative isolation methods could be envisaged [22]. An unbiased identification of cell surface proteins can be achieved by shotgun mass spectrometry after their selective enrichment [23–26]. Modern high resolution/sensitive instruments offering a much larger observation window now facilitate the identification of cell surface markers. In addition, several strategies have been developed for absolute (use of spiked-in standard peptides) or semi-quantitative quantifications. For semi-quantitative methods, labels have been introduced either chemically (isotope-coded affinity tag (ICAT) [27], isobaric tags for relative and absolute quantification (iTRAQ) [28], tandem mass tags (TMT) [29]) or metabolically (stable isotope labeling by/with amino acids in cell culture (SILAC) [30]). However, these labeling approaches suffer from imperfect labeling efficiencies, need for cell type-specific adaptation and, in case of SILAC a long cell culture, which limits this application for stem cell proteomics if one considers that their long term culture expansion can modify their cell surface proteome. During the past years, label-free methods have been developed [31] either based on protein coverage (number of identified peptides) or the number of MS^2^-spectra (spectral counts) [32] or chromatographic peaks areas [33]. These methods are also susceptible to changes in protein abundance, i.e. dynamic ranges and normalization methods [34–40] including absolute quantification. Normalization methods have been used to balance size differences of proteins or physicochemical differences of tryptic peptides facilitating a quantitative description of sample composition or sample-to-sample variations.

In the present study, we have evaluated the dynamic ranges of primary quantitative values and assessed the accuracy with which primary and derived normalized values reflect actual protein amounts. We have investigated the response of a modified t-test to address abundance changes, and we have demonstrated the power of this method by elucidating the composition of the DPSC surface proteome and its changes upon cell culture in either standard (S) medium containing 10% serum or basic expansion (BE) medium containing only 2% serum. Using this method we identified ≈400 plasma membrane proteins and quantified their changes during cell culture.

## Materials and Methods

### Antibodies

The primary antibodies (Abs) used for immunodetection are listed in S1 Table. The secondary Abs used for immunoblotting were horseradish peroxidase (HRP)-conjugated AffiniPure rabbit anti-mouse IgG and goat anti-rabbit IgG (Jackson Immunoresearch, West Grove, PA, USA); for immunocytochemistry, Cy3-conjugated AffiniPure goat anti-mouse IgG (H+L) and goat anti-rabbit IgG (H+L; Jackson Immunoresearch); and for flow cytometry, phycoerythrin (PE)–conjugated F(ab’)2 goat anti-mouse IgG and donkey anti-rabbit IgG (eBioscience, San Diego, CA, USA).

### Preparation of a yeast background proteome

1 g of baker's yeast was dissolved in 10 mL 4% SDS, 0.1 M DTT, 0.1 M Tris-HCl, pH 7.6. 250 U benzonase (Merck Millipore, Darmstadt, Germany) was added. Yeast cells were disrupted by 3 rounds of homogenization in an EmulsiFlex-C5 Homogenizer (Avestin, Ottawa, Canada) and cell debris was pelleted by centrifugation for 30 min, 10,000 x *g* at 4°C. The supernatant was filtered (0.45 μm) and kept on ice while the concentration of proteins was estimated. Aliquots of 80 μg total protein were used for filter-aided sample preparation (FASP). FASP was adapted from the Prince-Lab (http://openwetware.org/wiki/Prince:FASP). Dry proteins or protein pellets after recruitment assays were resuspended in 30 μl 4% (w/v) SDS, 0.1 M dithiothreitol (DTT), 0.1 M Tris-HCl, pH 7.6 and sonicated in an ultrasonic bath for 10 min at 65°C. The suspension was allowed to cool down to 20°C, mixed with 200 μl 8 M urea, 0.1 M Tris-HCl, pH 8.5 and transferred to a 30K filter insert (Vivacon500 V01H22, Sartorius, Göttingen, Germany) placed in a 2 ml Eppendorf tube. The solution was concentrated for 15 min in a table-top centrifuge at 14,000 x *g*, 20°C. Twice, 100 μl of 8 M urea, 0.1 M Tris-HCl, pH 8.5 was added and the sample was centrifuged for 15 min at 14,000 x *g* at 20°C. 100 μl 55 mM iodoacetamide in 8 M urea, 0.1 M Tris-HCl, pH 8.5 was added and alkylation was performed for 20 min at room temperature in the dark. The reagent was eliminated by centrifugation for 15 min at 14,000 x *g*, washed twice with 100 μL 8 M urea, 0.1 M Tris-HCl, pH 8.5 followed by two washes with 100 μl 50 mM ammonium bicarbonate. Digestion was performed by addition of 50 μL trypsin in a ratio of 1:40 (w/w) to the overall protein amount in 50 mM ammonium bicarbonate. The filters were vortexed and pulse-centrifuged to 2,000 x *g*, tubes closed and incubated for 4 h to 18 h at 37°C. The filter units were transferred into new collection tubes and centrifuged for 15 min at 14,000 x g. The filters were washed once with 40 μL 50 mM ammonium bicarbonate and the eluates acidified to 1% formic acid. Peptides were desalted using C18-SepPak cartridges (WAT023590, Waters, Milford, MA, USA). The cartridges were washed by one column volume (CV = 200 μl) of methanol, 1 CV of 80% acetonitrile, 0.1% trifluoroacetic acid and 3 CV of 0.1% trifluoroacetic acid. Peptides were loaded on top of the column material and slowly passed three times through it. The column was washed with 3 CV of 0.1% trifluoroacetic acid and eluted into a LC-vial with 1 CV of 80% acetonitrile, 0.1% trifluoroacetic acid. Peptides were dried in a speed-vacuum system and resuspended in 0.1% formic acid for 10 min in an ultrasonic bath.

### Preparation of dynamic range and accuracy standards

Tryptic digests of 500 pmol bovine serum albumin (#217498, Bruker, Germany) and 500 pmol ß-casein (#217507, Bruker) were each dissolved in 50 μl 0.1% formic acid by 15 min of sonication in an ultrasonic bath and combined. A dilution series of 500 amol and 5, 50 and 500 fmol and 5, 50 and 500 pmol digested proteins each in 90 μl 0.1% formic acid was prepared and replicates of 9 μl were measured in the LC-MS system. A similar dilution series was prepared with additional 600 ng of yeast peptides in each vial as a complex proteomic background and replicates of 9 μl were measured in the LC-MS system.

One vial of universal proteomics standard 2 (UPS2, Sigma-Aldrich, St. Louis, MO, USA) consisting of 49 proteins in different molar amounts (10.6 μg total protein) was reduced, alkylated and digested by FASP with 0.2 μg trypsin. One tenth of the total sample was used for each of four replicate LC-MS runs.

### Preparation of differential proteome standards

One vial of universal proteomics standard 1 (UPS1, Sigma-Aldrich) consisting of 49 proteins in an amount of 5 pmol (6 μg total protein) was reduced, alkylated and digested by FASP with 0.1 μg trypsin. This stem solution and a preparation of a yeast background proteome were used to construct a series of samples as follows (actual protein amounts injected into the LC-MS system, each sample contained 60 ng yeast): 20 fmol, 6.7 fmol, 2.2 fmol, 0.74 fmol, 0.24 fmol UPS1. All samples were prepared in quadruplets.

### Cell isolation and culture

DPSCs from impacted third molars of healthy young donors (17–23 years, n = 10) undergoing tooth extraction for orthodontic reason were described previously [14, 15]. The Ethical Committee of the Faculty Hospital in Hradec Králové, Czech Republic approved the study to J.K. Tissues of adult donors were acquired under written informed consent directly, in case of individuals below 18 years, who gave their assent to donation, legally authorized persons (parents) provided written informed consent on their behalf. Removed pulp tissue was digested with collagenase (0.2 mg/ml; Sevapharma, Prague, Czech Republic) and dispase (2 mg/ml; Thermo Fisher Scientific, Rockford, IL, USA) for 70 min at 37°C, mechanically dissociated and filtered through a 70-μm cell strainer (BD Biosciences, Franklin Lakes, NJ, USA).

Pulp cell suspensions were cultured either in a basic expansion medium (BE medium), which consists of minimum essential medium alpha modification (α-MEM; Thermo Fisher Scientific), 50 nM dexamethasone (Sigma-Aldrich), 0.2 mM ascorbic acid 2-phosphate (Sigma-Aldrich), 2 mM L-Glutamine (Thermo Fisher Scientific), 100 U/ml penicillin and 100 μg/ml streptomycin (Thermo Fisher Scientific) supplemented with 2% fetal calf serum (FCS; PAA Laboratories, Linz, Austria), 10 ng/ml human recombinant epidermal growth factor (EGF; PeproTech, London, UK) and 10 ng/ml human recombinant platelet-derived growth factor (PDGF-BB; PeproTech) or standard medium (S medium) consisting of α-MEM medium, 0.2 mM ascorbic acid 2-phosphate, 2 mM L-Glutamine, 100 U/ml penicillin and 100 μg/ml streptomycin supplemented with 10% FCS. Media were changed after 2–3 days. First passage was done when single colonies appeared (i.e. after 5–10 days), and afterward cells were trypsinized upon reaching 70% confluence. Cells were split 1 to 3.

### Cell surface biotinylation

DPSCs (2.5 × 10^7^ cells) from both culture conditions (2 versus 10% FCS) were biotinylated as described previously [26].

### Sample preparation, mass spectrometric analysis and protein identification

We followed the same procedure as described previously [26]. In brief, the biotinylated proteins were fractionated in a Tris-glycine PAGE gel and stained with Coomassie G-250. Each lane was cut into 24 slices. The proteins embedded in each slice were reduced with DTT, alkylated with iodoacetamide, digested overnight with trypsin in a 1:50 ratio and subjected to LC-MS/MS analysis. Peptides were separated on an EASY-nLC HPLC system (Proxeon, Odense, Denmark) equipped with a fused silica microcapillary C18 column (Proxeon, length 10 cm; inner diameter 75 μm; particle size 3 μm, 100 Å pore size. The gradient used was: A, 0.1% formic acid; B, acetonitrile, 0.1% formic acid with a final concentration of 80% B. Mass spectrometry analysis was made on an LTQ Orbitrap XL mass spectrometer (Thermo Fisher Scientific). The MS data were analyzed using the Proteome Discoverer 1.0 software (Thermo Fisher Scientific). Mascot (version 2.2.2) and the SwissProt database (SwissProt_56.9.fasta) were used for interpretation of spectra applying the following settings: The taxonomy was set to human and trypsin as the enzyme allowing up to two missed cleavages. Precursor mass tolerance was set to 10 ppm, fragment mass tolerance to 0.5 Da. As a static modification carbamidomethylation (of Cysteine) was chosen and as dynamic modifications deamidation (of Asparagine and Glutamine) and oxidation (of Methionine). Protein hits were filtered for a minimum of identified peptides of two with a minimum score of 40, possessing either transmembrane domains and known plasma membrane localization or a signaling sequence and known lipid modification. Five independent experiments were performed to determine the total proteome of DPSCs grown under the two different conditions, whereas three independent experiments were performed to determine the cell surface proteomes of the DPSCs cultured in standard or basic expansion medium, respectively.

### Processing of mass spectrometry spectra by MaxQuant

All raw-files from XCalibur were loaded into MaxQuant (V1.2.2.5) [33] and an experimental table was created in a way that all slices of one lane were assigned to the same experiment. Carbamidomethylation of cysteine was considered as a fixed modification and oxidation of methionine, acetylation of the N-terminus, and deamidation of asparagine or glutamine as variable modifications. Other parameters are multiplicity of 1, trypsin as enzyme, maximum number of modifications per peptide of 3, maximal missed cleavages of 2, maximum charge of 4 with individual peptide mass tolerances allowed. A suitable FASTA data base was assigned (UNIPROT-TrEMBL 2012–01). Parameters in the protein identification tab were as follows: peptide and site FDR of 0.01, maximum peptide posterior error probability of 1, all minimum peptides of 1, filter labeled amino acids disabled, second peptides, re-quantify, label-free quantification and match between runs (min 2) enabled.

### Parameters of label-free mass spectrometry

Quantification using peptide numbers (PN), exponentially modified protein abundance index (emPAI), spectral counts (SC), normalized spectral abundance factors (NSAF), MaxQuant intensities (MQ), Label-free quantification intensities (LFQ), extracted ion intensity protein abundance index (xPAI), and intensity-based absolute quantification values (iBAQ) are collected or calculated as follows and demonstrated on the prepared and measured accuracy standards (UPS2): PN were extracted from the respective columns of the MaxQuant proteinGroupfile output, emPAI were calculated for each protein (p) from the PN and the number of observable peptides (PO, see below: iBAQ) as follows:

$$emPAI_{p}=10^{\left(\frac{PN_{p}}{PO_{p}}\right)}$$

SC were extracted from the MS/MS-count columns of the MaxQuant proteinGroupfile output, NSAF of single proteins (p) were calculated from the SC, the length of the respective protein (L) and the number (n) of proteins in the data base as follows:

$$NSAF_{p}=\frac{\left(\frac{{SC}_{p}}{L_{p}}\right)}{\sum_{i=1}^{n}\left(\frac{SC_{i}}{L_{i}}\right)}$$

MQ were extracted from the respective columns in the proteinGroups.txt file, LFQ were extracted from the respective columns of the MaxQuant proteinGroupfile output, xPAI were calculated using the ‘Precursor Ions Area Detector’ module of the Proteome Discoverer 1.2 Software (Thermo Fisher Scientific) with a mass range window of 2 ppm, iBAQ were calculated from the MQ-intensities in the proteinGroups.txt file by division through the number of observable peptides and logarithmizing. Numbers of theoretically observable peptides were calculated using an in-house python script (S2 Method) after retention time limits have been estimated by a yeast proteome analysis and the use of another python script (S1 and S3 Methods). Parameters that restrict observability are extreme hydrophobicity or hydrophilicity, charge states and too low or high number of amino acids and can be passed to the python programs via a setting.ini–file (S4 Method). Since implementation into MaxQuant (Version 1.2.2.5) iBAQ values were extracted from the respective columns in the proteinGroups.txt file. This streamlined the workflow, but restricted the so far adjustable parameters to the preset values of 6 to 35 amino acids in peptide length. All values were paired in an SQL-database and further analyzed in the statistical computing and graphics software R.

### Differential proteomics: Permutation-based t-tests on LFQ intensities

The proteinGroups.txt-file was loaded into Perseus [41] with all LFQ-intensities as ‘expression’. All proteins matching any of the three categorical annotations (1: Only identified by site, 2: Reverse, 3: Contaminant) representing unreliable spectrum matches or supposed contaminants were deleted, LFQ-intensities were logarithmized, experiments were grouped to sample or control and proteins that were not detected in all three replicates of at least one group were deleted. Missing values were imputed by normal distribution with parameters that were adapted using histogram plots. A two sided t-test was performed with a false discovery rate between 0.1 and 1%, and a slope value of 0.2 to 1, the control was hereby assigned to ‘group 2’. The protein table and the threshold curve table were exported for further analysis in R.

### Gene ontology assignment and enrichment analysis

Proteins in the proteinGroupfile.txt-output of MaxQuant were sorted for their sum intensities in a decreasing order and bins of 50 proteins were created. Uniprot identifiers were pasted into the gene products-field of the term enrichment tool of AmiGO (http://amigo.geneontology.org/cgi-bin/amigo/term_enrichment). Like for microarray analysis, no background set was selected. The database filter was set to UniProt KB and electronically inferred (IEA) annotations were allowed. The Bonferroni-corrected p-value threshold was set to 0.05 and the minimal number of gene products to 2. This procedure was applied for all intensity bins, the tables of GO terms and associated p-values were exported, unified in an SQL database and further analyzed in R.

### Reverse transcription polymerase chain reaction

Total RNA was prepared from DPSCs using an RNeasy Mini Kit with column DNAase treatment according to the manufacturer’s instructions (Qiagen, Hilden, Germany). cDNA template was synthesized using a High-Capacity cDNA Reverse Transcription (RT) Kit (Applied Biosystems, Foster City, CA, USA) from 500 ng of total RNA in a 20-μl reaction volume. Three independent preparations were prepared. Polymerase chain reaction (PCR) was performed using Platinum Taq DNA polymerase (Thermo Fisher Scientific) as follows: 28 (in the case of CADH2, EGFR, GAPDH, ITA8, ITA10, MFGM) or 32 (LRC15, NCAM2, SLIK2, uPAR) cycles consisting of 45 s denaturation, 94°C; 45 s annealing, 61°C; 75 s extension, 72°C; with an initial denaturation step for 5 min, 94°C and final extension for 10 min at 72°C. The primer pairs used are listed in S2 Table. 5-μl aliquots of the PCR products were analyzed using agarose gel (1.7%) electrophoresis.

### Protein extraction and immunoblotting

Cells were washed and scraped in ice-cold PBS, followed by their solubilization in RIPA buffer (1% NP-40, 0.5% deoxycholate, 0.1% SDS, 150 mM NaCl, 50 mM Tris-HCl, pH 8.0) in the presence of 1 mM phenylmethylsulfonyl fluoride and complete protease inhibitors for 30 min at 4°C. After centrifugation (10 min, 16,000 × *g*), the protein concentration in the supernatant was determined using a BSA protein assay (Thermo Fisher Scientific). Three independent protein preparations were prepared. Proteins (25 μg) were separated by SDS-PAGE (7.5%) and transferred to a PVDF membrane (pore size 0.45-μm, Merck-Millipore). Membranes were incubated at 4°C overnight in blocking buffer (PBS containing 5% low fat milk powder and 0.3% Tween 20) and then probed with a given primary Ab (S1 Table). Antigen-Ab complexes were detected using the appropriate HRP-conjugated secondary Ab and visualized with enhanced chemiluminescence developing reagents (GE Healthcare Life Sciences). Membranes were generally exposed for 1–10 min to Hyperfilm (GE Healthcare Life Sciences), and quantified after scanning using ImageJ software.

### Immunocytochemistry

#### Cell surface labeling

Cell surface labeling was performed as described previously [14]. Briefly, cells grown on polyornithine/fibronectin-coated glass coverslips were washed with PBS followed by ice-cold Ca/Mg-PBS, blocked in immunofluorescence buffer (Ca/Mg-PBS containing 0.2% gelatin) for 10 min and then incubated with primary Abs (S1 Table) diluted in the same buffer for 30 min at 4°C. As negative control, the primary Ab was omitted. Unbound antibodies were removed by five washes with ice-cold immunofluorescence buffer. Fixative, 4% paraformaldehyde (PFA) in PBS, was added at 4°C and coverslips were placed for 30 min at room temperature. The fixative was removed by three washes with PBS, and the residual PFA was quenched with 50 mM NH_4_Cl for 10 min. Cells were then incubated with Cy3-conjugated anti-mouse or anti-rabbit secondary Ab for 30 min at room temperature. After washing in PBS, nuclei were counterstained with 4'-6-diamidino-2-phenylindole (DAPI; Sigma-Aldrich), coverslips were rinsed sequentially with PBS and distilled water and mounted in Mowiol 4–88 containing the anti-fading agent 1,4-diazobicyclo-[2.2.2]-octane (DABCO) (Merck-Millipore). Samples were examined using a BX61 Olympus microscope equipped with an Olympus DP71 digital camera, and the images were prepared using Adobe Photoshop and Illustrator software.

#### Intracellular labeling

Cells grown on glass coverslips were washed three times in PBS and fixed with 4% PFA in PBS for 30 min at room temperature. After thorough washing with PBS, residual PFA was quenched with 50 mM NH_4_Cl for 10 min. Samples were incubated in blocking buffer with 0.2% gelatine and 0.2% saponin for 20 min at room temperature and then incubated without (negative control) or with anti-N-cadherin Ab (see S1 Table) directed against an intracellular epitope, for 30 min at room temperature. Afterward, labeled cells were processed as described above.

### Flow cytometry

DPSCs were harvested either by trypsin/EDTA treatment for 4 min at 37°C or by scraping (in the case of uPAR/CD87 and MFGM detection). After inactivation of trypsin and centrifugation (5 min at 300 × *g*), cells were resuspended in PBS containing 1% bovine serum albumin and 100 μl-cell suspension aliquots were incubated with either PE-conjugated or unconjugated primary Abs (see S1 Table) for 30 min at 4°C. In the latter case, incubation with species-specific PE-conjugated secondary Ab was performed. After washing with PBS, 10,000 (or 30,000 in the case of scraped cells) events were acquired on an LSRII flow cytometer (BD Biosciences). Instrument settings and gating strategies were established using the appropriate isotype Ab control or secondary Ab alone. Data were analyzed using FlowJo software (TreeStar, Ashland, USA). Median fluorescence intensity (MFI) was calculated as a difference of MFI values obtained from stained and negative control (i.e. with isotype primary Ab or secondary Ab alone) cell populations.

## Results

To evaluate the proteomic composition of different dental pulp stem cell (DPSC) samples and the differences between them, we first set out to establish a comprehensive label-free mass spectrometric platform based on the most accurate mass spectrometric parameters.

### Dynamic range of basic mass spectrometric parameters

**Fig 1:**
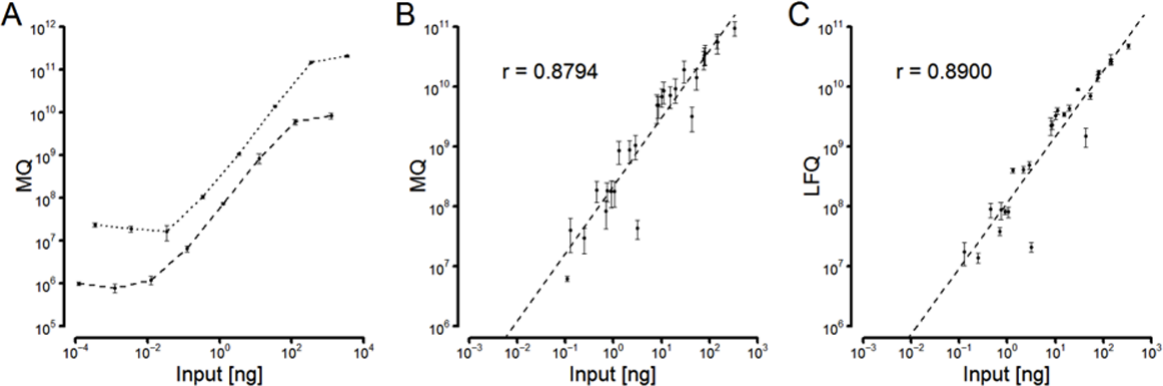
Dynamic range of intensity values. The different intensity parameters were calculated as indicated in Materials and Methods. **A.** Dynamic range of intensity values estimated by MaxQuant (MQ) using BSA (dotted line) and β casein (dashed line) as standard. **B.** Accuracy of MQ values using the Universal proteomic standard (UPS) 2 proteins. **C.** Accuracy of label-free quantification values (LFQ), a normalization of MQ-values established in B.

First, we compared the dynamic ranges of the mass spectrometric parameters i.e. spectral counts (SC), MaxQuant intensities (MQ), and extracted ion intensity protein abundance index (xPAI), using a dilution series of a mixture of albumin and β casein. We found that the best dynamic and linear range achieved by the MQ intensity was covering five orders of magnitude with respect to mass input (Fig 1A and S1 Fig). The dynamic range of the SC method was clearly inferior for both albumin and β casein. The experiment was repeated in the presence of a yeast background proteome, which affected the three parameters unequally. In the SC or xPAI method, the detection of albumin appeared unaffected, whereas that of β casein was restricted to high amounts. Clearly, the outperforming parameter was MQ with a linear range over five orders of magnitude for both proteins while having both curves nearly superposed.

### Linear ranges of normalized parameters

Second, we wanted to assess the accuracies and linear ranges of normalized parameters to estimate protein abundances of a broader spectrum of standard proteins. We used a tryptic digest of 49 proteins presented in a range from 0.5 to 50,000 fmol (Universal proteomic standard UPS2) and calculated the values of the following parameters: peptide numbers (PN), exponentially modified protein abundance index (emPAI), spectral counts (SC), normalized spectral abundance factors (NSAF), MaxQuant intensities (MQ), label-free quantification intensities (LFQ), extracted ion intensity protein abundance index (xPAI) and intensity-based absolute quantification values (iBAQ) (Fig 1B and 1C and S2 Fig). The quantitative indices differ not only in the overall correlation (correlation coefficients), but also in the magnitude of single outliers, which would lead to false estimations of single protein abundances. This was particularly the case for emPAI and PN, but also for LFQ, where a single protein was underestimated by two orders of magnitude. PN and emPAI proved to correlate poorly, when no manual data deselection was allowed. All other parameters appeared to be suitable for assessing proteome compositions. Interestingly, the magnitude of single outliers was diminished when the parameters were compared to the mass inputs rather than to the common molar inputs enlightening another level of normalization. Also, the overall correlation was enhanced in a similar way. The two most eligible indices were LFQ and iBAQ. The latter appeared as an almost ideal index for protein abundance in a sample with low standard deviations of repeated measurements, an excellent correlation coefficient over a huge dynamic range and outliers below one order of magnitude. On the other hand, LFQ demonstrated to be an accurate measurement. Therefore, we decided to proceed with this parameter to assess its use in the comparison of different samples.

### Assessment of LFQ values

Finally, we assessed label-free quantification intensity (LFQ) values for their applicability in comparing different samples. Hubner *et al*. [41] used a modified t-test, meanwhile implemented in their Perseus software. However, an exact algorithm of this t-test had not been published and an objective choice of threshold parameters remained unclear. We set out to study the responsiveness of this test and its threshold parameters. For this purpose, we used the universal proteomic standard 1 (UPS1) consisting of 49 equimolar proteins and spiked it into a yeast background proteome in a series of different amounts (S3 and S4 Figs). Four technical replicates of all such samples were analyzed via LC-MS and protein identification was accomplished using MaxQuant. LFQ values were log2-transformed, and proteins were eliminated if not at least 3 values out of four replicates were achieved. A permutation based modified t-test, similar to a significance analysis of microarray experiments, was conducted using the MaxQuant Perseus software, with a threshold value of 0.01 and a slope value of 1.0. No protein was found to be significantly different when 0.74 versus 0.24 fmol UPS1 proteins were compared, since it was impossible to yield three LFQ values as required for any of the proteins with an input of only 0.24 fmol. As expected, the number of quantifiable proteins increased with the amounts spiked in. In contrast, the accuracy of estimation of ratios increased with the actual ratio, but not with increasing input amounts. No saturation of this increase was reached within the experiments while even small (3-fold) changes in a complex background proteome were elucidated. No meaningful increase in false positives occurred when the threshold value was raised to 0.25 (from common values between 0.001 to 0.05) testifying an excellent robustness of the underlying algorithm.

### Cell surface proteome of dental pulp stem cells

**Fig 2:**
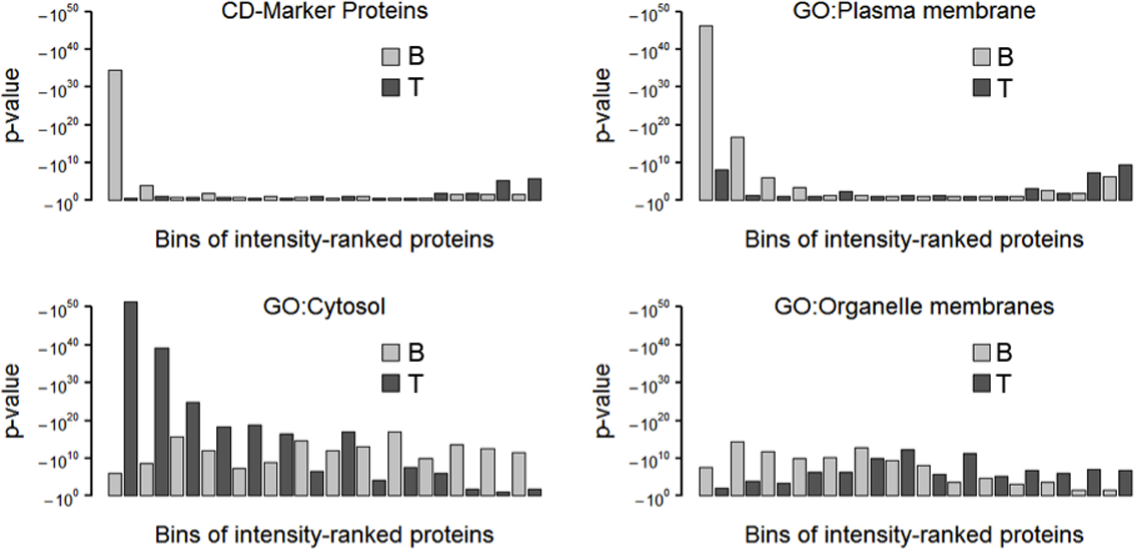
Enrichment analysis. The total proteome (T) and biotinylated, affinity-purified cell surface proteins (B) were prepared from DPSCs cultured in standard medium and analyzed by MS as indicated in Materials and Methods. The proteins were ranked and binned by decreasing intensity (abundance). In each bin, the number of proteins with a gene ontology-assignment to CD markers, plasma membrane, cytosol or organelle membrane was estimated. On the ordinate, -log_10_(p-values) represent the frequency of occurrence of the respective gene ontology normalized for its explanatory power.

We applied the resulting workflow (S5 Fig) for the analysis of the cell surface proteomes of stem cells isolated from dental pulp and subsequently cultivated either in basic expansion (BE) medium or in standard (S) medium [14]. We compared their total proteomes (T(BE) and T(S)) with those obtained after cell surface biotinylation and affinity enrichments (B(BE) and B(S)). Because many proteins can bind non-specifically to the bead matrix used for affinity purification, we prepared control samples from cells whose cell surface proteins were not labeled with biotin (C(BE) and C(S)). We then identified the proteins present in this set of samples fractionated by SDS-PAGE (S6 Fig and S3–S5 Tables). We first ascertained the efficiency of the enrichment procedure. Common Venn diagrams were plotted for all proteins and proteins associated with gene ontology to either the plasma membrane or cytosol or organelle membranes (S7 Fig). This resulted in a 40% overlap of all proteins and nearly the same number of proteins identified either in any cell surface protein-enriched (B) or total proteome (T) sample. We got a 47% overlap for the proteins assigned to organelle membranes and slightly more proteins (30%) for the cell surface protein-enriched samples alone than for the total proteome (23%). The overlap of cytosolic proteins was also 47% but prevailing in the total proteome alone (43%) as compared to the exclusive cell surface protein-enriched (10%). More importantly, for the plasma membrane proteins the overlap was 38% with 46% proteins for cell surface protein-enriched samples and only 16% identified in the total proteome samples. A cell surface protein-enrichment procedure is expected to yield more than just 3 times surplus of plasma membrane proteins, but the procedure increases not only the number of different cell surface proteins but also the amount. The traditional Venn diagrams and also pie-diagram representations disregard intensities and hence individual protein abundances restricting the focus of enrichment analyses to frequency alterations. Hence, we developed a graphical analysis based on Fisher’s exact tests of bins of intensity-ranked proteins (Fig 2 and S1–S4 Methods). For each bin, the p-value represents the probability of the number of the identified proteins assigned to the respective gene ontology to occur by chance. We use the negative logarithm of the p-value for the ordinate axis to have a huge bar representing enrichment of that gene ontology in the bin. In these graphs, proteins assigned to organelle membranes distributed equally over all bins of the samples enriched in cell surface proteins and almost alike in the total proteome. Cytosolic proteins also distributed equally over all bins of the cell surface protein-enriched sample but were highly enriched in the higher intensity bins of the total proteome samples. Proteins assigned to the plasma membrane were only enriched in the first bin of the total proteome samples but highly in the first third of the bins of the enriched cell surface proteins. This type of analysis is clearly an improvement over Venn diagrams particularly when high sensitivity mass spectrometry is carried out and high numbers of low intense proteins distort the results.

We identified 100 CD-markers (S3 Table) in the samples enriched in cell surface proteins and only 36 in the total proteome sample. 286 CD-marker proteins were not identified in our samples. We combined gene ontologies in a Boolean expression (S5 Method) to set up a list of identified proteins that we expected to represent true positive cell surface proteins, while we found that gene ontology assignments to cell surface were not sufficient as a criteria. Identified proteins were hereby accepted as cell surface proteins if they are GPI-anchored (GA) or transmembrane proteins (TM) assigned to the plasma membrane (PM) or if they are signal peptide-containing proteins (SP) that are assigned to either the extracellular space (ECS), region (ECR) or matrix (ECM). This resulted in 365 proteins identified in the cell surface protein-enriched samples (55 high intense, 125 intense, 172 moderate intense, 13 low intense) and 118 proteins identified in the total proteome samples (21 high intense, 58 intense, 38 moderate intense, 1 low intense) with an overlap of 96 proteins (S4 Table). Spearman's rank correlation coefficient of overlapping proteins is 0.183 illustrating a high bias of the chemical enrichment procedure. Consequently, while cell surface protein enrichment is useful to sensitively detect cell surface proteins, quantitative proteome composition has to be inferred from total proteome data. Cell surface protein enrichment should be used to assess relative differences of the same proteins in two different samples and to set up a list of negative CD-markers. 254 (66%) of the 387 listed proteins were found to be significantly different from the background binders in t-tests of B(S) vs. C(S) or B(BE) vs. C(BE). The t-tests chose 582 of 1930 proteins as significant from background with a threshold of 0.001 (slope = 0.2). There is no explicit way to set the threshold and hence the size of the list is a matter of choosing the threshold. 89% of the proteins selected by the Boolean expression prevailed in B(S) vs. C(S) and 59% of the t-test-significant hits are part of this list as well as 35% of the CD markers. Further manual analysis of this significant hits yielded only 3.6% of the Boolean and significant hits being false positives. The assumption of this analysis is that, while t-testing on biotinylated versus control samples can be used to discriminate proteins that occur on the cell surface, the selected proteins must be further validated by a Boolean expression to eliminate non-cell surface proteins, like cytosolic proteins bound to integral membrane proteins.

**Fig 3:**
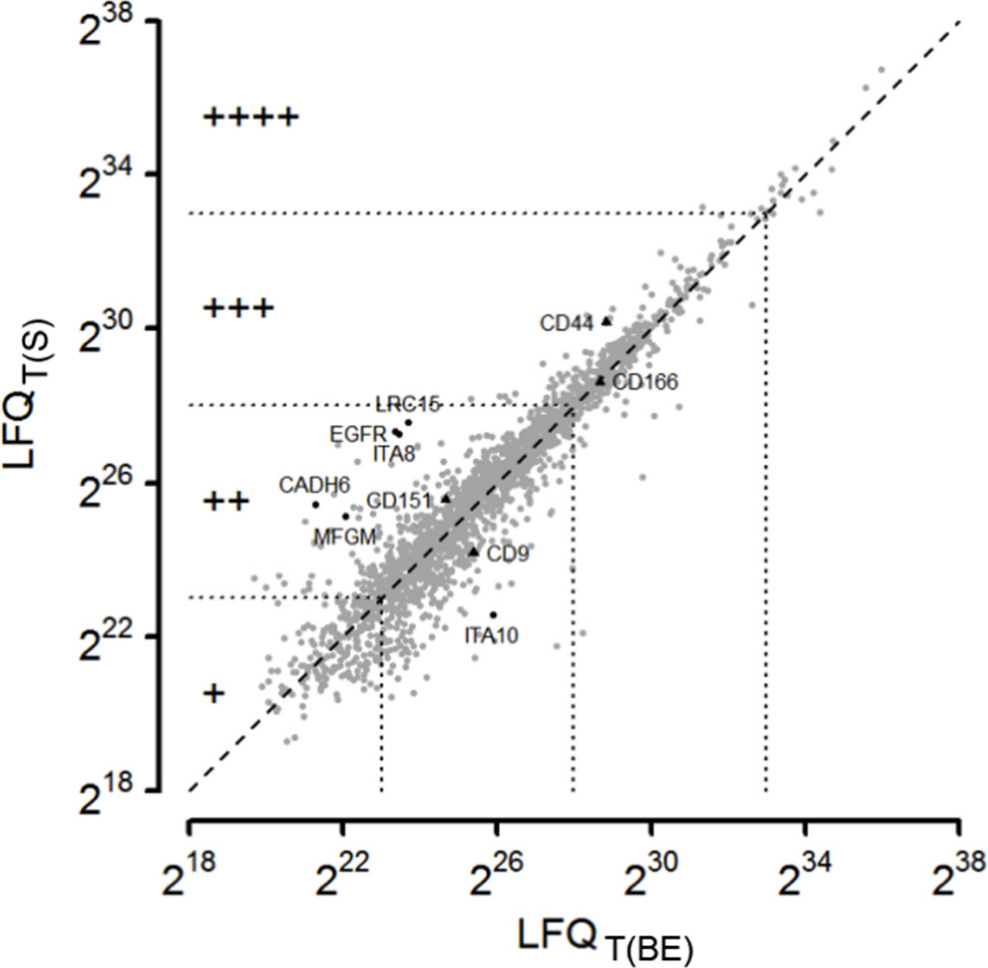
Compositional analysis of the total proteome. Averages of LFQ-intensities of total proteins from DPSCs cultured in basic expansion (BE) or standard media (S) were compared. Intensity regions are defined by equidistant border lines (2^23^, 2^28^, 2^33^) dividing identified proteins into groups of low abundance (+, roughly 1/6 of all proteins), moderate abundance (++, 2/3 of all proteins), high abundance (+++, 1/6 of all proteins) and the 1% most intense proteins (++++). Grey dots represent average values of triplicates. Plotting standard deviations in both directions would be unreadable. Instead, a modified t-test was used to reveal proteins that significantly differ between both sample types (black dots). The compositional analysis was used to select marker proteins supposed to be positive for validation experiments (black triangles) along with two marker proteins (CD39, CD106, not indicated in the graph), which were only identified upon cell surface proteome enrichment.

To assess the relative abundance of cell surface markers, we analyzed the sample composition from averages of normalized intensity values (LFQ) of the total proteome samples (Fig 3). The log2-transformed LFQ intensities were simplified by dividing into five abundance classes: below 18, 18 to 23, 23 to 28, 28 to 33, and above 33 (labeled as: -, +, ++, +++, ++++, respectively). Proteins found to be significantly differentially expressed in a differential proteome analysis (see below) can be seen in the total proteome offside the bisecting line (ITA10, EGFR, ITA8, CADH6, MFGM) (Fig 3). Seven proteins were not identified suggesting a general low abundance (UPAR, CADH2, CBPM, 1A24, NCAM2, GPNMB, LSAMP). Several proteins appear in higher distance to the bisecting line although they proved not to be significant in the differential proteome analysis. This is either due to a lack of enrichment or by a high standard deviation in the estimation of LFQ_T(BE)_ or LFQ_T(S)_.

The common MSC markers are proteins enabling prolonged self-renewal, stemness factors (Nanog, Oct 3/4, Sox 2), and cell surface markers [10, 14]. Table 1 compares these respective cell surface markers with our identified proteins. Stro-1 was omitted because the nature and identity of the associated antigen is unclear. We found 7 of 8 proposed MSC markers in high or very high intensities in the samples enriched in cell surface proteins (B) and 5 of them in the total proteome sample (T). CD271 (also known as p75NTR) was identified in neither of the samples. It remains to be analyzed, if its absence is a result of the isolation or cultivation methods used. Nonetheless, the lack of CD271 is in agreement with a recent report indicating that a CD34-negative subpopulation of DPSCs does not express CD271 [42]. None of the hematopoietic cell surface markers (e.g., CD34, CD45, CD133) were identified indicating high purity of the stem cell isolation. The cell surface markers that we did not identify are also of importance because they can be used as negative selection markers. We did not identify 286 protein markers of the CD classification in the total proteome or cell surface protein-enriched samples (S3 Table). In addition to the expected MSC surface markers, we identified 31 CD-markers in the total proteome samples (one was found exclusively here) and 73 CD-markers in the cell surface proteome enriched samples. Beside the CD-classified proteins for which commercial antibodies for flow cytometry are generally available, we identified 79 intense or high intense potential cell surface protein markers in the total proteome samples and 180 in the cell surface proteome enriched samples. These results were in absolute agreement with our previous flow cytometry and immunocytochemistry studies on these samples [14].

**Table 1:** Stem cell surface markers.

Marker	Acc. No.	B	T	Name
Mesenchymal stromal cell markers				
CD29	P21926	++++	+++	CD29 antigen
CD44	P16070	++++	++++	CD44 antigen
CD73	P21589	++++	++++	5-nucleotidase
CD90	P04216	++++	+++	Thy-1 membrane glycoprotein
CD105	P17813	++++	-	Endoglin
CD146	P43121	+++	-	Cell surface glycoprotein MUC18
CD166	Q13740	++++	++++	CD166 antigen
CD271	P08138	-	-	Tumor necrosis factor receptor superfamily member 16
Hematopoietic (stem) cell markers				
CD34	P28906	-	-	Hematopoietic progenitor cell antigen CD34
CD45	P08575	-	-	Receptor-type tyrosine-protein phosphatase C
CD117	P10721	-	-	Mast/stem cell growth factor receptor Kit
CD133	O43490	-	-	Prominin-1

B,T: Average of LFQ intensities of cell surface proteome in enriched (B) or total proteome (T) samples.

**Fig 4:**
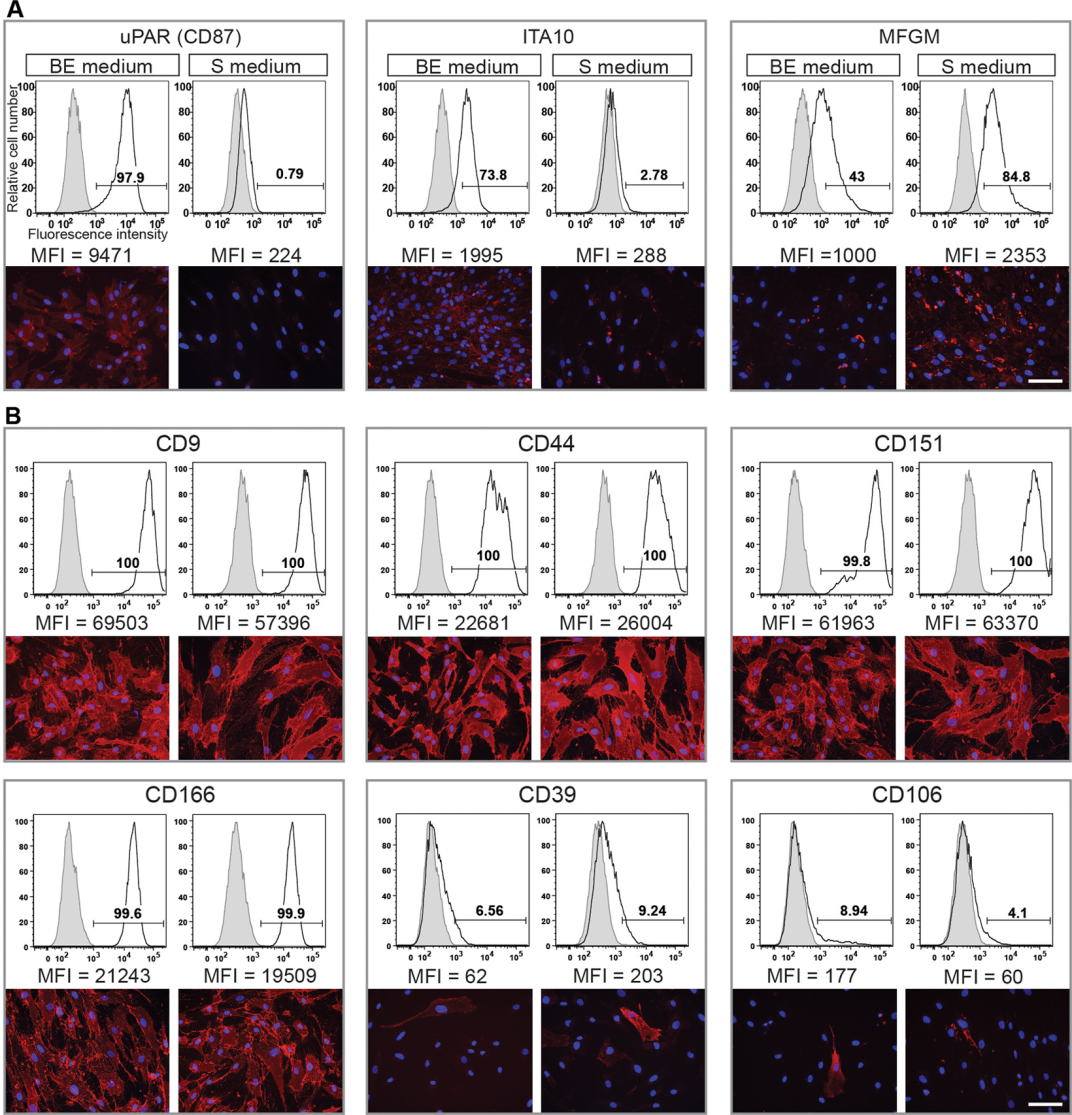
Cell surface expression of MS-based identified surface proteins by DPSCs cultured in different media. **A and B.** Flow cytometry (top panels) and indirect immunofluorescence (bottom panels) analyses of various MS-based identified markers derived from DPSCs cultured either in basic expansion (BE) or standard (S) media. Cells were obtained from the same donors (i.e. donors 1 and 6) as used in the MS analyses. Cell surface markers that displayed a differential expression by MS or not are depicted in panels A and B, respectively. For flow cytometry, the antigen expression (open area) and the appropriate isotype-matching or secondary Ab control (grey filled area) are depicted. The median fluorescence intensity (MFI) is indicated. For indirect immunofluorescence, nuclei were visualized with DAPI. Representative flow cytometry histograms and fluorescent images from three independent experiments are displayed. Scale bar, 50 μm.

As shown in Fig 4, six CD-markers reflecting different intensity regions in the total proteome were selected for validation: CD44 and CD166 (high intense), CD151 and CD9 (moderate intensity) and CD39 and CD106, which were only identified after enrichment of the cell surface proteins (Table 2). All 4 CD-markers that were identified in the total proteome were proven to be equally suited for flow cytometry and showed positive staining in immunocytochemistry. The CD-markers that were identified only after enrichment of cell surface proteins were negative in flow cytometry and heterogeneously expressed among cells.

**Table 2:** Selected CD-markers.

CD	Acc. No.	B	T	Name
CD44	P16070	++++	++++	CD44 antigen
CD166	Q13740	++++	++++	CD166 antigen
CD9	P21926	++++	+++	CD9 antigen
CD151	P48509	++++	+++	CD151 antigen
CD39	P49961	++	-	Ectonucleoside triphosphate diphosphohydrolase 1
CD106	P19320	++	-	Vascular cell adhesion protein 1

B,T: Average of LFQ intensities of all cell surface proteome enriched (B) or total proteome (T) samples.

### Differences in the cell surface proteomes of dental pulp stem cells grown in different media

**Fig 5:**
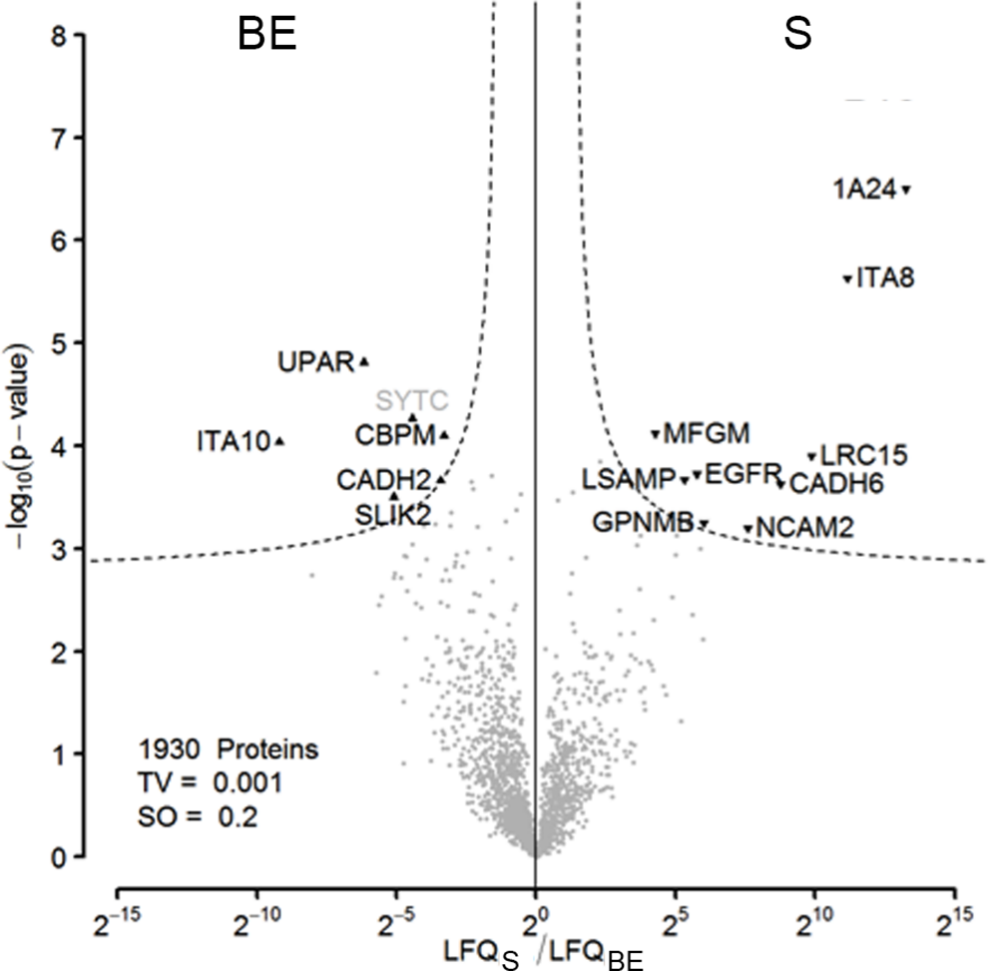
Differential proteome analysis. Proteins were isolated after cell surface biotin labeling of DPSCs isolated from a single donor and cultured in either basic expansion (BE) or standard media (S). 9 proteins were passing a conservative threshold of 0.001 on the S side and 6 showed preferential abundance in the BE samples. Black labels: Significant hits, assigned to the cell surface proteome by the Boolean algorithm. Grey labels: Significant non-cell surface proteome proteins.

Next, we analyzed the difference in the cell surface proteome of cells growing in the basic expansion (BE) medium and standard (S) medium (Fig 5). Using t-testing on LFQ intensities of the cell surface protein-enriched samples, we identified 6 proteins enriched on cells grown in BE medium when compared to cells grown in S medium and 9 proteins enriched on cells grown in S medium when compared to cells grown in BE medium. We did not filter out background proteins in advance, as a proteomic background is mandatory for this type of modified t-test. 4 out of the 6 proteins identified on cells grown in BE medium (UPAR, ITA10, CADH2, CBPM) and 8 of the 9 proteins identified on cells grown in S medium (1A24, ITA8, MFGM, EGFR, CADH6, NCAM2, GPNMB, LSAMP) were assigned to the plasma membrane by the Boolean algorithm.

### Validation of MS data by RT-PCR and immunoblotting

**Fig 6:**
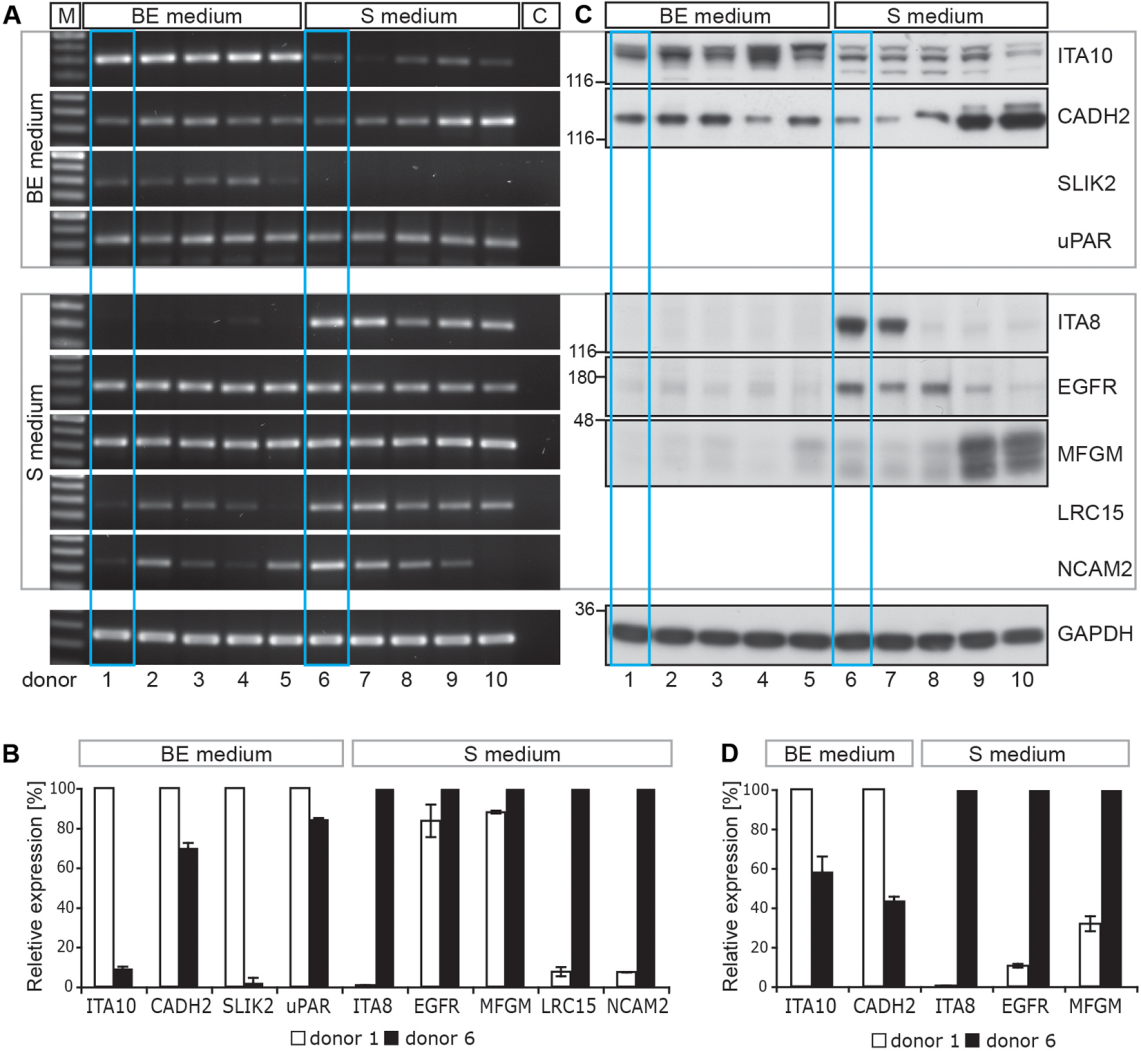
Differential expression of MS-based identified surface proteins by DPSCs cultured in different media. RT-PCR (**A and B)** and immunoblotting (**C and D)** analyses revealed the expression level of selected MS-identified cell surface markers of DPSCs cultured either in basic expansion (BE) or standard (S) media. Samples from 5 independent donors from both culture conditions were analyzed (donors 1–5 and 6–10, respectively). MS data were derived from cells obtained from donors 1 and 6 (blue frame). The markers at the top were found by MS to be predominantly expressed on cells growing in BE medium, whereas the others were mainly found after culture in S medium. Glyceraldehyde 3-phosphate dehydrogenase (GAPDH) was used as loading control. **A.** M: 100 base pair DNA Ladder, c: Negative control (i.e. without cDNA template) **C.** The positions of prestained molecular mass markers are indicated on the left. **B and D.** Expression level of specific cell surface markers from donors 1 and 6 was quantified. Data are the mean of two to three independent experiments using distinct mRNA and protein preparations; bars indicate the variation of the individual values from the mean. The expression level was arbitrarily set to 100% for the marker predominantly identified under a specific culture condition. Note that the expression of protein markers enriched in DPSCs cultured in S medium fluctuates between donors as illustrated by CADH2 and MFGM (see also S8 and S9 Figs).

RT-PCR and immunoblotting experiments confirmed that the two selected groups of MS-based identified cell surface markers were indeed differentially expressed (Fig 6 and S9 Fig). For instance, ITA10, CADH2 and SLIK2 were enriched on cells cultured in basic expansion (BE) medium, whereas ITA8, LRC15 and NCAM2 were enriched on cells growing in standard (S) medium. Likewise, EGFR and MFGM (MFGE8, lactadherin) showed a notable differential expression by immunoblotting (Fig 6C and 6D), but not by RT-PCR (Fig 6A and 6B), in agreement with the MS data (Table 3). The low level of EGFR protein, but not its transcript, in cells cultured in BE medium could be explained by the presence of EGF (10 ng/ml), which could promote the endocytosis, and hence its degradation [43]. MFGM is a secreted protein that interacts with alpha-V-beta-3 and alpha-V-beta-5 integrins (ITGB3 and ITGB5; [44]). The two latter proteins were nevertheless not differentially expressed by cells cultured in either BE or S media as demonstrated by the MS data (Table 3) suggesting indirectly that MFGM might interact with a novel unidentified component preferentially expressed in cells isolated and cultured in S medium.

**Table 3:** Summary of the validation of the differential proteome analysis of DPSCs, cultivated in two different media by RT-PCR and immunoblotting of total proteomic samples.

Protein	Acc. No.	CSP	WB	RT-PCR	Name
Differential MS-analysis: BE>S					
UPAR	Q03405	+		0	Urokinase plasminogen activator surface receptor
ITA10	O75578	+	a	a	Integrin alpha-10
CBPM	P14384	+			Carboxypeptidase M
CADH2	P19022	+	h	0,h	Cadherin-2
SYTC	P26639	-			Threonine—tRNA ligase, cytoplasmic
SLIK2	Q9H156	+		a	SLIT and NTRK-like protein 2
Differential MS-analysis: S>BE					
1A24	P05534	+			HLA class I histocompatibility antigen, A-24 alpha chain
ITA8	P53708	+	a,h	a	Integrin alpha-8
CADH6	P55285	+			Cadherin-6
NCAM2	O15394	+		h	Neural cell adhesion molecule 2
EGFR	P00533	+	a,h	0	Epidermal growth factor receptor
MFGM	Q08431	+	a,h	0	Lactadherin
LSAMP	Q13449	+			Limbic system-associated membrane protein
GPNMB	Q14956	+			Transmembrane glycoprotein NMB
LRC15	Q8TF66	+		a,h	Leucine-rich repeat-containing protein 15

CSP: Cell surface protein, BE: Basic expansion medium, S: Standard medium, WB: Western blotting, RT-PCR: Reverse transcription-polymerase chain reaction. a: Affirmed difference, h: Heterogeneity between donors, 0: No difference detected, c: Conflictive difference (none).

### Validation of MS data by flow cytometry and immunocytochemistry

Although the level of uPAR (CD87) transcript did not show a significant difference between culture conditions as observed by RT-PCR (Fig 6A and 6B), its analysis at the protein level using flow cytometry and immunocytochemistry revealed a weak and homogenous expression in cells cultured in BE medium, but not in S medium (Fig 4A) in agreement again with the MS data. These protein-based detection techniques also confirmed that ITA10 and MFGM were predominantly expressed in cells cultured in BE and S media, respectively (Fig 6A). As revealed by the MS data, CD9, CD44, CD151, CD166 were strongly expressed at the cell surface of DPSCs isolated and cultured under both experimental conditions (Fig 4B). In contrast, CD39 and CD106 were expressed only in subpopulation of cells (Fig 4B), which explains their poor recovery by MS (S3 Table).

Finally, it is important to note that heterogeneity in terms of expression levels of given markers among donors was mostly observed in cells isolated and cultured in standard (S) medium. In addition to CADH2 and MFGM (Fig 6C), we could observe by immunoblotting that CD146 expression fluctuated considerably between donors (S8A and S9 Figs; e.g., donors 6–8 by comparison to 9–10). Immunocytochemistry confirmed their fluctuations among donors (S8B and S9 Figs) suggesting that the culture condition, where 10% of serum is added may favor the isolation and growth of various progenitor cell populations as recently suggested [14, 45]. Moreover, the phase contrast microscopy revealed that cells derived from donors 6 and 9 differed in their growth pattern (S8C Fig). For instance, those derived from donor 9 grew in tight clusters, in contrast to the ones from donor 6 as well as donor 1, the latter being cultured in basic expansion (BE) medium. The cellular heterogeneity was also confirmed by a differential expression of cytoskeleton constituents such as actin and tubulin (S8A and S9 Figs). The threonine-tRNA ligase SYTC is believed to be a false positive finding. Although the t-test threshold was set very strictly, so that the false discovery rate could be assumed far below 1% false positives can never be completely excluded. However, increasing the number of replicates could aid. No conflictive results were yielded from the validation experiments. For a generalization of the findings, a higher number of donors should be used, preferably for flow cytometry or immunoblotting.

## Discussion

Several label-free approaches have been developed and based either on intensity values or on indirect parameters subsequently normalized in various, different ways. To implement a label-free mass spectrometry platform, we first performed a systematic comparison of label-free mass spectrometric parameters (Fig 1 and S1 Fig). We found that the MaxQuant (MQ) intensity performed best in terms of dynamic range and linearity over four orders of magnitude, even in the presence of a complex proteomic background. When we assayed the accuracy using standard proteins (S2 Fig), the MQ-intensity and its normalized values, the label-free quantification intensity (LFQ) and the intensity-based absolute quantification values (iBAQ) were clearly superior to the purely engineered values, peptide number (PN), spectrum counts (SC), exponentially-modified protein abundance index (emPAI) and normalized spectral abundance factor (NSAF). These experiments also showed that the accuracy was even better, when the values were compared to mass amounts instead of molar amounts. Moreover, outliers were minimized allowing an even more reliable absolute quantification of all proteins tested. In a detailed view, iBAQ values performed slightly better than LFQ values, and both were clearly dominating compared to pure MQ values. However, there is a basic difference between iBAQ and LFQ values. LFQ-values are normalized in a way that reduces sample-to-sample variations, thus allowing the comparison of different samples. To achieve this with iBAQ values, the values have to be further normalized with spiked-in UPS standards as it was first demonstrated in Schwanhäusser *et al*. [40]. Therefore, we used LFQ values to estimate protein abundance in both compositional analyses of single samples and differential proteome analyses of two or more samples. While we cannot completely exclude single principle outliers through atypical physico-chemical effects, when different proteins are compared within a sample, comparing different groups of proteins seems justifiable. This is for instance the case when the success of a given biochemical enrichment procedure has to be demonstrated.

**Fig 7:**
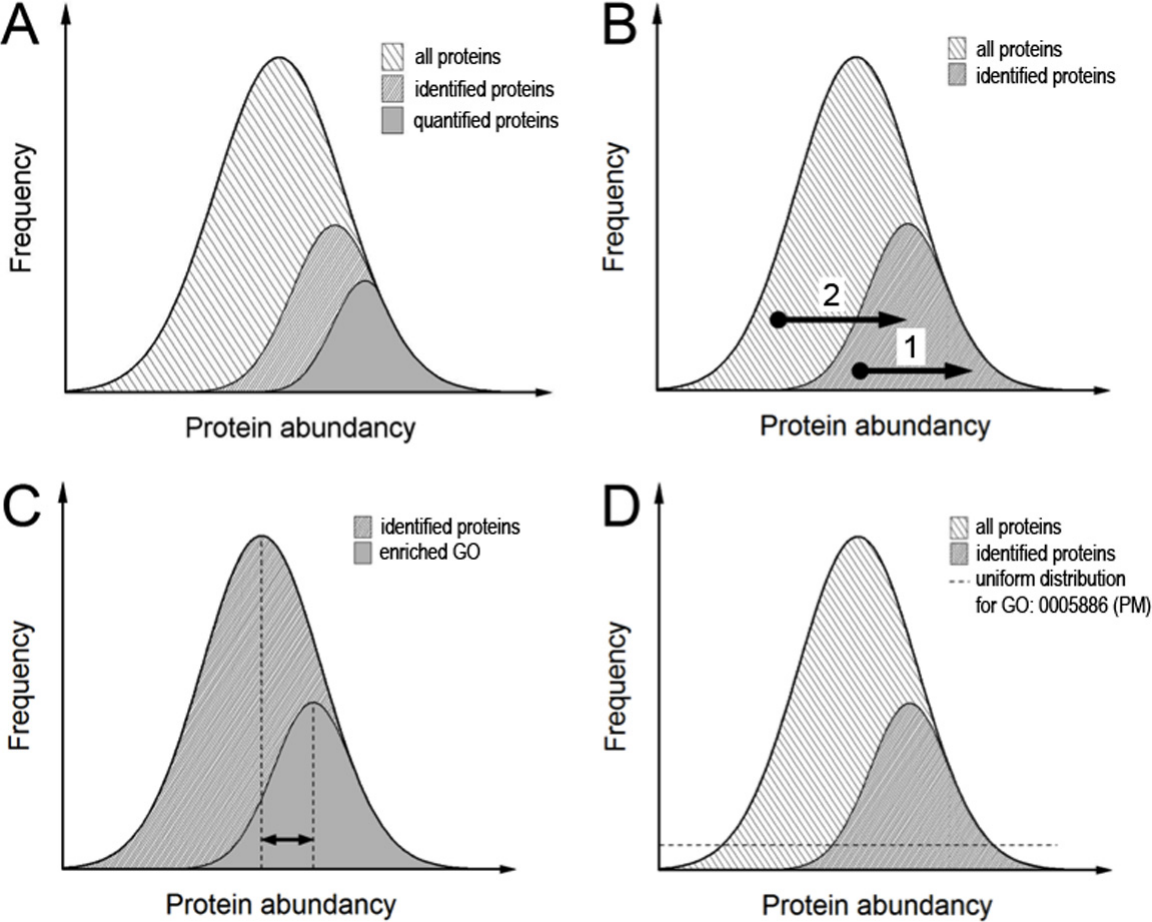
Histograms to assess protein enrichment. The proteins were grouped for their abundances and the relative frequency with which different proteins in the abundance bins occurred. **A.** Bias of detection. In shotgun proteomic experiments not all proteins present can be detected. The subpopulation of identified proteins is composed of more abundant proteins than the average of all proteins present. Likewise, the subpopulation of quantified proteins is biased to the more abundant proteins among the identified proteins. The reason for the prevalence to identify abundant proteins is that abundant proteins are most likely yielding more intense peptide precursors and are hence most likely selected for fragmentation. Likewise, abundant proteins are most likely generating more and higher scoring peptide identifications, explaining the bias (modified from [52]). **B.** Observation of enrichment. Upon enrichment, proteins can be shifted either within the observation window (1) or into another (2). Moreover, proteins that are not promoted by the enrichment procedure can be decreased in intensity and even leave the observation window. **C.** Enrichment analysis comparing the mean intensity of a subpopulation of identified proteins with the overall mean. This leads to information about the sample composition but as a chemical enrichment procedure does not necessarily lead to such an intrasample shift of the intensity distribution the method’s axiom seems improper. As an example, if an absolute enrichment had been achieved leading to the identification of proteins from a specific gene ontology only, this method would yield no enrichment at all. **D.** Schematic representation of all 20,256 proteins annotated in UniProt/Sprot and the 22.4% of these, which were assigned as plasma membrane proteins (PM). As there was no prior information about abundances in the data bank, p-value-based enrichment calculations assumed a uniform distribution, which was very unlikely to reflect the real situation. The strategy presented here was to calculate p-values for cell surface proteome enriched samples as well as for total proteome samples and compare p-values of respective intensity bins.

The typical shotgun approach uses a TopN-program that conducts the mass spectrometer to automatically select the N most intensive peptides in the MS1 spectrum to be fragmented for MS2. As the most abundant proteins are more likely generating higher intense MS1 peaks, they are more likely being identified. This results in a bias for the identification of the most abundant proteins in a proteomic sample. Not all of the proteins that are identified can be quantified. The reasons for this are manifold and differ in the quantitative methods. In shotgun experiments, low intense proteins are often not identified in replicates and are hence not selected because statistical tests are mandatory. This results in a further bias for the selection of the most abundant of the identified proteins to be quantified as well (Fig 7A).

Enrichment procedures like we have used here for cell surface proteins aim to increase the relative abundance of proteins of interest and either elevate those proteins into the observation window or increase the intensity within all the hitherto identified proteins (Fig 7B). A question that arises is how to define and detect enrichment success. Pie charts are broadly used to demonstrate sample composition reflecting the relative numbers of proteins assigned to certain gene ontologies. All parts of this pie should add up to 100%. However, as proteins are assigned to many different gene ontologies there are redundant occurrences of proteins in different groups. Also, there is no information telling if the size of a group reflects enrichment or just a probabilistic sampling. Pie charts reflect the detection of proteins and do not take into account the abundance of different proteins. The latter is also the case for Venn diagrams, which are commonly used to compare the number of proteins assigned to a specific group between two samples. With highly sensitive mass spectrometers, proteomes are more broadly covered and it becomes more difficult to distinguish a sample and a control in a Venn diagram. Cox and Mann [46] use a ranking of proteins by intensity in a histogram plot and define enrichment as a subpopulation of proteins sharing a specific gene ontology with a mean value deviating from the mean of all other proteins. This is similar to Pan *et al*. [47], where the subpopulation mean is compared to the mean of all proteins identified (Fig 7C). This approach has its weakness when proteins of a specific GO-assignment enter the observation window in a number that could not have happened by chance, but do not deviate in their distribution mean from the overall. For instance, if an absolute enrichment had been prepared leading to the identification of proteins from specific gene ontology only, this method would yield no enrichment at all. Cox and Mann [46] expanded their approach to a two dimensional form which allowed comparisons of different samples and even from different types of experiments. They converted the distance from the mean into a ranking value and prepared a multivariate analysis of variance (MANOVA). In that way, the approach still relies on intrasample distributions and does not implicate comparisons with the ‘gene universe’ [48], i.e. the entry of proteins into the observation window.

Our approach is based on intensity-ranked even-sized contingents of proteins (‘bins’), where accumulation of certain gene ontologies is tested for their likelihood to occur by chance. We define enrichment as an increase in the negative decadic logarithm of the resulting p-value, and replace the simple number of proteins in histogram plots herewith. This enables different gene ontologies to be compared as GO categories weighed for their explanatory power. A problem that arises with such an approach is that we do not know the actual intensity distribution of a given gene ontology within all proteins. If for instance, gene ontology for the glycolytic pathway is studied, the assumption can be made that the involved proteins generally occur in high copy numbers. Without prior information about all the protein abundances, one has to assume a uniform distribution for the calculation of the p-values (Fig 7D). The problem is circumvented in an extended two-dimensional form of the approach by comparing ranked intensity bins of samples after chemical enrichment and total proteomic samples. The approach comprises both the proteins that were entering the identification window and those that were intensified inside due to an intrinsic ranking comparison.

Compared to our previous shot-gun experiment identifying ~200 cell surface proteins of MSCs isolated from bone marrow [26], we have here identified ~400 cell surface proteins of DPSCs making it to our knowledge the most comprehensive analysis to date. We confirmed the expression of the CD markers, CD97, CD112, CD239, CD276 and CD316, which we identified as novel MSC markers in our earlier study, and in addition, we found several new ones. Notably, several proteins belonging to the tumor necrosis factor receptor superfamily (CD40, CD120a, CD261, CD262, CD264, and CD266), different integrins (alpha-4, alpha-6, and alpha-10), or interleukin receptors (CD121a, CD130, CD213a1, CD217, and CDw210b) were identified. Previously, we did not detect CD56 as an MSC marker [49], but now we were able to identify it in the enriched DPSC samples. Nonetheless, some proteins previously identified as MSC markers (e.g. integrin alpha-11) [26, 50] were not detected here, suggesting either a very low level of expression or different subpopulations or a difference in MSCs isolated from dental pulps versus bone marrow.

Interestingly, there are a few, but significant, changes in the proteomes of DPSCs cultured in either low (BE medium) or high (S medium) serum content milieu. Four out of the five proteins differentially expressed with a high expression in cells cultured in BE media, were identified as novel markers. They include CD87 (UPAR), integrin alpha-10, CBPM and SLIK2. Since most of the previous studies were performed using medium containing 10% serum, this could explain why they were not identified before. However, CD325 (CADH2, N-cadherin), another marker with a high expression in BE medium, was identified previously [26, 51]. Five of the nine markers with a higher expression after culture in S medium are also novel markers for MSCs and include integrin alpha-8, CADH6, NCAM2, GPNMB and LRC15. The HLA class 1 histocompatibility antigen, A-24 was of course identified due to the fact that we used different donors for the different conditions. As mentioned above, there are donor-donor differences in both growth pattern and surface marker expression in DPSCs cultured in S medium. This is probably due the fact that cells after long-term culture especially with high serum levels start to differentiate spontaneously [17, 18] suggesting that it is more preferable to use BE medium for isolation and expansion of DPSCs.

In conclusion, we established label-free quantitative mass spectrometric workflows for compositional and comparative proteomic analyses. Applied to dental pulp stem cells, these approaches demonstrated their excellent suitability for the unbiased detection of potential stem cell surface markers. With this method, we were able to achieve a very comprehensive cell surface proteome of DPSCs from single donors, as we have identified and quantified ≈400 plasma membrane proteins and quantified their changes during cell culture.

## Supporting Information

S1 Fig Dynamic range of quantitative values. **A.** Two-component mixtures of different amounts of albumin (dotted) and β casein (dashed) were trypsin-digested and analyzed via LC-MS. Highest dynamic range over five orders of magnitude along with a strong linearity was achieved by the use of MaxQuant intensity values (MQ) and similar results were achieved by using the extracted ion chromatogram-based protein abundance index (xPAI). The spectral count index (SC) was restricted to a dynamic range over three orders of magnitude in case of the smaller β casein. Using mass amounts on the abscissa normalized the data for protein length and converges both curves. **B.** Dynamic range in a complex proteomic background. Albumin (dotted) and β casein (dashed) in different amounts were spiked into a total yeast proteome, trypsin-digested and analyzed via LC-MS. Sample complexity slightly reduced the dynamic range of all values. The MaxQuant intensity performed best in terms of dynamic range and linearity over four orders of magnitude.
(TIF)

S2 Fig Accuracy of protein amount estimations. The linearity of different normalized parameters was compared to a broad range of spike-in amounts of Universal proteomic standard (UPS) 2 proteins. PN and its normalized derivative emPAI were highly affected by large outliers. SC, its normalized derivative NSAF and all intensity-based parameters were generally suited for quantitative analyses. All parameters showed slightly better Pearson correlation coefficients when they were opposed to the mass inputs suggesting another level of normalization. Some proteins deviated largely from the regression lines, hampering confident estimation of single protein amounts, whereas assumptions over group contingents like proteins sharing certain gene ontology seemed to be justified. This was not the case for iBAQ values, which suited best also in overall correlation. Here, deviations of single values were less than one order of magnitude from the regression line. LFQ intensities were only slightly less accurate and as they were constructed as a normalization between samples, they were consequently used for further differential proteome analyses.
(TIF)

S3 Fig Volcano plots for differential proteome analysis. Different amounts of UPS1 standard proteins (black dots) were spiked into a total yeast proteome (grey dots). To assess the response of the model to protein abundance, changes in each volcano plot show four replicates, where two of these samples were compared. The dashed lines represent significance thresholds (FDR = 0.01 and s_0_ = 1.0). Numbers inside the plot indicate spike-in amounts of USP1 that were compared. L: Lowest concentration, H: Highest concentration.
(TIF)

S4 Fig Boxplots of ratios of UPS1 proteins normalized to the actual input ratios. The analyses (experiment labels) are related to S3 Fig. Numbers inside the plot assign the number of proteins passing the significance thresholds. The accuracy increases with higher input ratios but not with increasing input amounts. Even small changes in a complex background proteome are elucidated.
(TIF)

S5 Fig Experimental workflow. DPSCs were cultured either in basic expansion medium (2% FCS with PDGF and EGF additives) or in standard medium (10% FCS) (indices BE or S, respectively). **A.** Total proteome (samples T(BE) and T(S)). **B.** Control samples without labeling but with affinity purification (samples C(BE) and C(S)). **C.** Cell surface proteome-enrichment with biotinylation and affinity purification (samples B(BE) and B(S)).
(TIF)

S6 Fig Comparison of gel lanes. **A.** Gel lanes from the total proteome samples were indistinguishable in their band patterns. **B.** Gel lanes of the controls exhibited small differences. **C.** Arrows indicate some obvious differences in the gel band patterns of cell surface proteome-enriched samples. **D.** Uncropped gel images. Lanes used for A, B and C and cutting patterns are indicated.
(TIF)

S7 Fig Venn diagrams of DPSC proteomes. Venn-diagrams of cell surface proteome-enriched (B) and total proteome (T) samples. Around 40% of the 2,867 identified proteins were found in both types of samples. The cell surface proteome-enriched sample exhibited an approximately three fold higher number of proteins with a gene ontology assignment to plasma membrane localization (GO:0005886), whereas the total proteome sample contained four times more cytosol-localizing proteins (GO:0005829). Proteins assigned to organelle membranes (GO:0031090) were not preferentially enriched in either of the samples.
(TIF)

S8 Fig DPSCs isolated and cultured in standard medium display heterogeneity among the donors. **A**. The expression profile of proteins acquired from DPSCs of 10 independent donors and cultured either in basic expansion (BE) or standard (S) media as indicated was examined by immunoblotting. Glyceraldehyde 3-phosphate dehydrogenase (GAPDH) was used as loading control. The position of prestained molecular mass markers is indicated on the left. In addition to CD146, note the variation in actin and α-tubulin among donors (e.g. donors 9 and 10 by comparison to others) suggesting indirectly an alteration in the cellular morphology. **B and C.** DPSCs from donors 1, 6 and 9 were either cell surface labeled (CD146, MFGM) or fixed, permeabilized and labeled (CADH2) prior analysis by (**B**) fluorescence microscopy or observed by (**C**) phase contrast microscopy. Nuclei were visualized with DAPI. Representative immunoblots and microscopy images from two to three independent experiments are displayed. Scale bars, 50 μm.
(TIF)

S9 Fig Uncropped images/scans of agarose gels and immunoblots shown in Fig 6 and S8 Fig. The relevant information presented in Fig 6A and 6C and S8A Fig are indicated in red boxes. DNA ladders and the molecular weight markers are presented on the left. Dashed line indicates the cut of PVDF membrane in 2 segments that were incubated with distinct primary antibodies.
(PDF)

S1 Method Python-script to facilitate ascertaining of retention time limits. (DOCX)

S2 Method Python-script to estimate numbers of observable tryptic peptides. (DOCX)

S3 Method Parameter-file for listing I (RCLimits.ini). (DOCX)

S4 Method Parameter-file for listing II (Settings.ini). (DOCX)

S5 Method Boolean expression to create a list of and to filter for cell surface proteins. (DOCX)

S1 Table List of primary antibodies used for immunodetection. (DOCX)

S2 Table List of primers used for RT-PCR analysis. (DOCX)

S3 Table CD-marker proteins identified. (DOCX)

S4 Table Non CD-marker cell surface proteins identified. (DOCX)

S5 Table Proteins identified in the different MS analyses of different donors. Included are a protein description, the Uniprot accession string, the Uniprot accession number, the numbers of peptides identified and the posterior error probabilities (PEP) provided by MaxQuant used as an identification quality indicator, similar to a score value. Razor and unique peptides and LFQ intensity values are also shown. Column A (Only identified by site), Column B (Reverse), Column C (Contaminant) are categorical classifications by MaxQuant used to filter proteins out. Five independent experiments were performed to determine the total proteome of DPSCs grown under the two different conditions, whereas three independent experiments were performed to determine the cell surface proteomes of the DPSCs cultured in standard or basic expansion medium, respectively.
(XLSX)
