# Supplementary figures and images for: Cell Surface Proteome of Dental Pulp Stem Cells Identified by Label-Free Mass Spectrometry
Source: PLoS One. 2016 Aug 4;11(8):e0159824. doi: 10.1371/journal.pone.0159824 (PMC4973913; doi:10.1371/journal.pone.0159824)

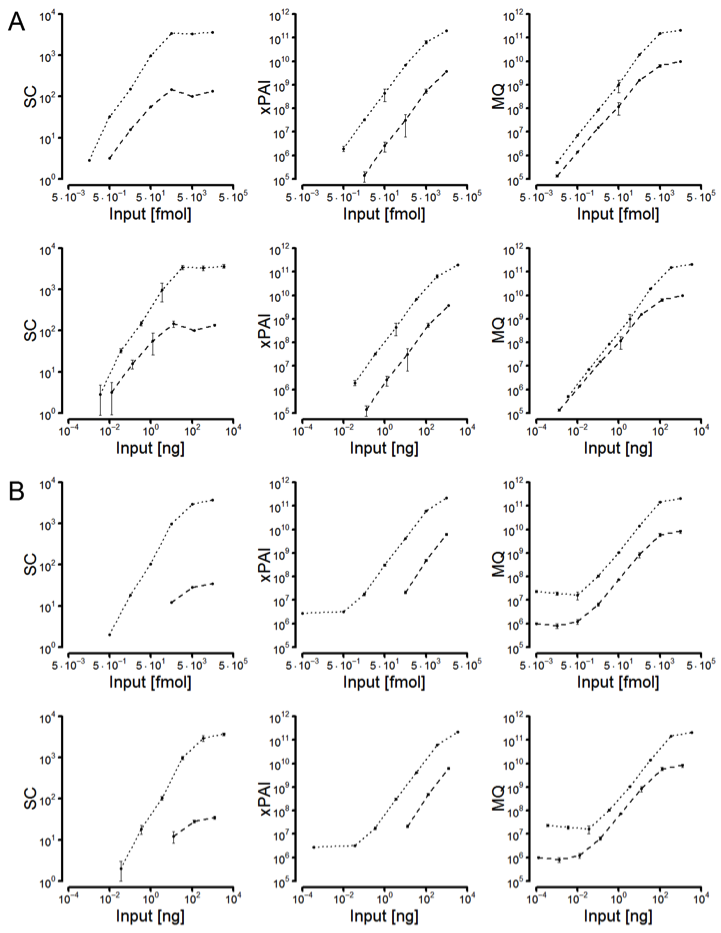

Supplement: S1 Fig — A. Two-component mixtures of different amounts of albumin (dotted) and β casein (dashed) were trypsin-digested and analyzed via LC-MS. Highest dynamic range over five orders of magnitude along with a strong linearity was achieved by the use of MaxQuant intensity values (MQ) and similar results were achieved by using the extracted ion chromatogram-based protein abundance index (xPAI). The spectral count index (SC) was restricted to a dynamic range over three orders of magnitude in case of the smaller β casein. Using mass amounts on the abscissa normalized the data for protein length and converges both curves. B. Dynamic range in a complex proteomic background. Albumin (dotted) and β casein (dashed) in different amounts were spiked into a total yeast proteome, trypsin-digested and analyzed via LC-MS. Sample complexity slightly reduced the dynamic range of all values. The MaxQuant intensity performed best in terms of dynamic range and linearity over four orders of magnitude. (TIF) [file pone.0159824.s001.tif]

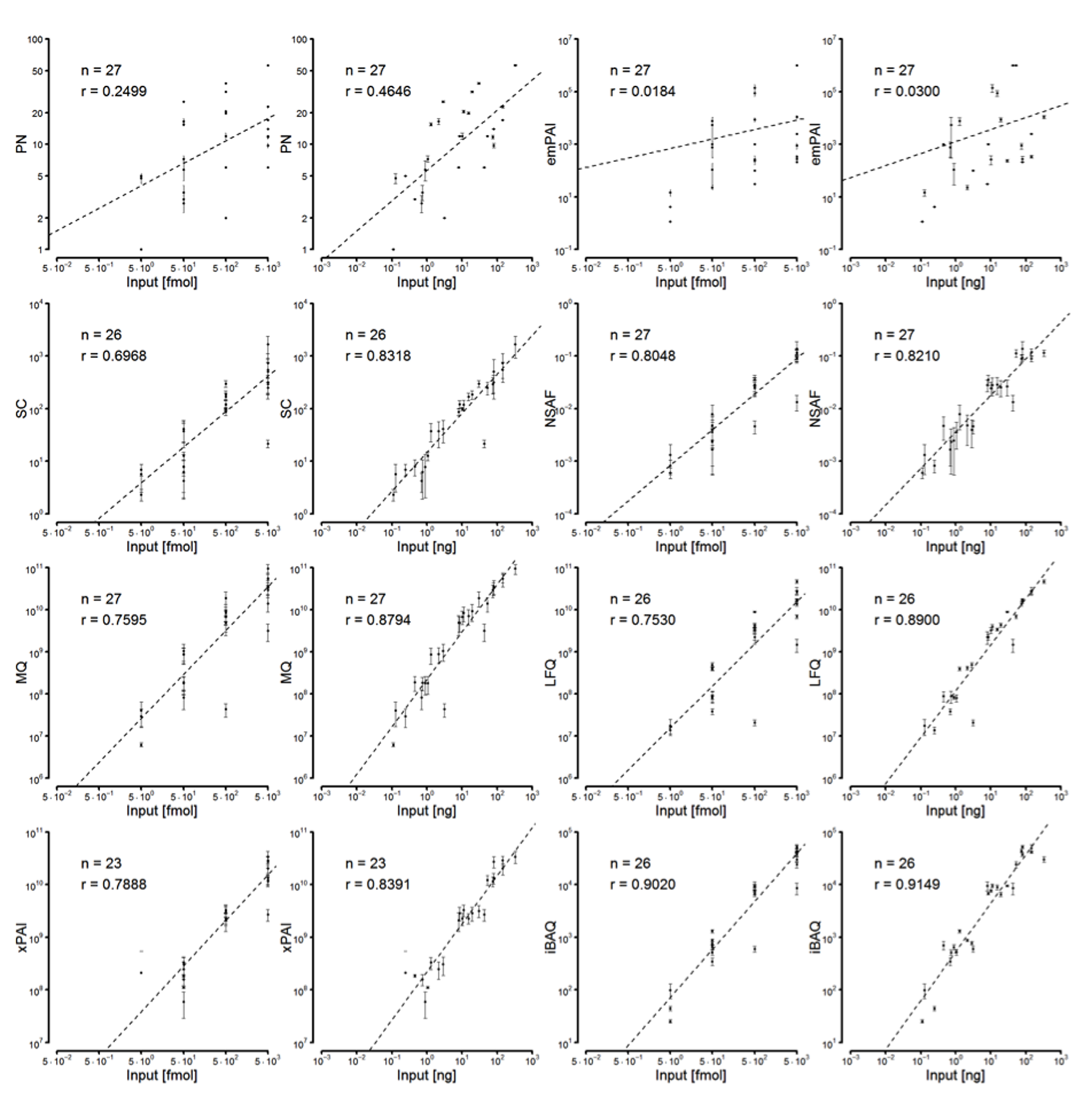

Supplement: S2 Fig — The linearity of different normalized parameters was compared to a broad range of spike-in amounts of Universal proteomic standard (UPS) 2 proteins. PN and its normalized derivative emPAI were highly affected by large outliers. SC, its normalized derivative NSAF and all intensity-based parameters were generally suited for quantitative analyses. All parameters showed slightly better Pearson correlation coefficients when they were opposed to the mass inputs suggesting another level of normalization. Some proteins deviated largely from the regression lines, hampering confident estimation of single protein amounts, whereas assumptions over group contingents like proteins sharing certain gene ontology seemed to be justified. This was not the case for iBAQ values, which suited best also in overall correlation. Here, deviations of single values were less than one order of magnitude from the regression line. LFQ intensities were only slightly less accurate and as they were constructed as a normalization between samples, they were consequently used for further differential proteome analyses. (TIF) [file pone.0159824.s002.tif]

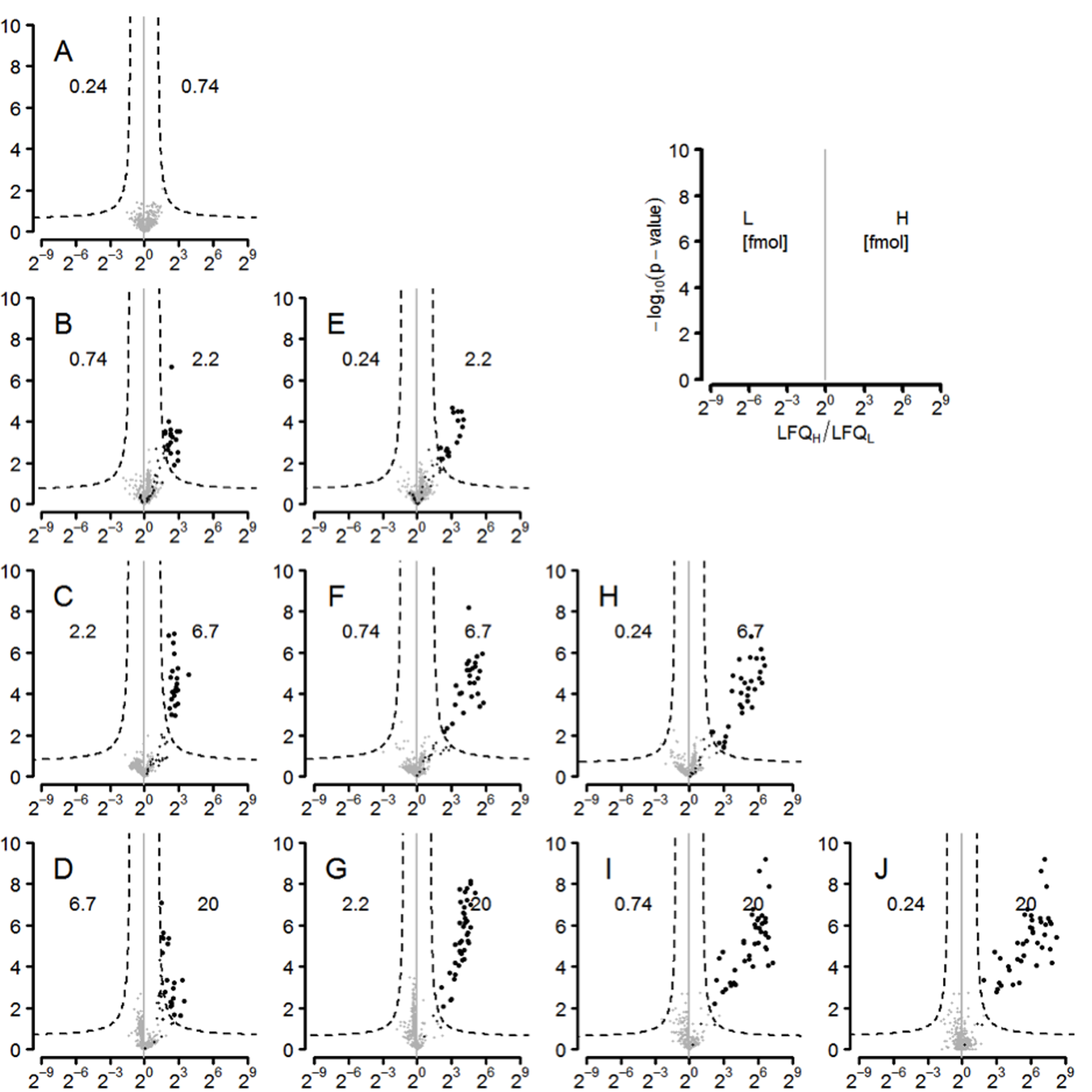

Supplement: S3 Fig — Different amounts of UPS1 standard proteins (black dots) were spiked into a total yeast proteome (grey dots). To assess the response of the model to protein abundance, changes in each volcano plot show four replicates, where two of these samples were compared. The dashed lines represent significance thresholds (FDR = 0.01 and s0 = 1.0). Numbers inside the plot indicate spike-in amounts of USP1 that were compared. L: Lowest concentration, H: Highest concentration. (TIF) [file pone.0159824.s003.tif]

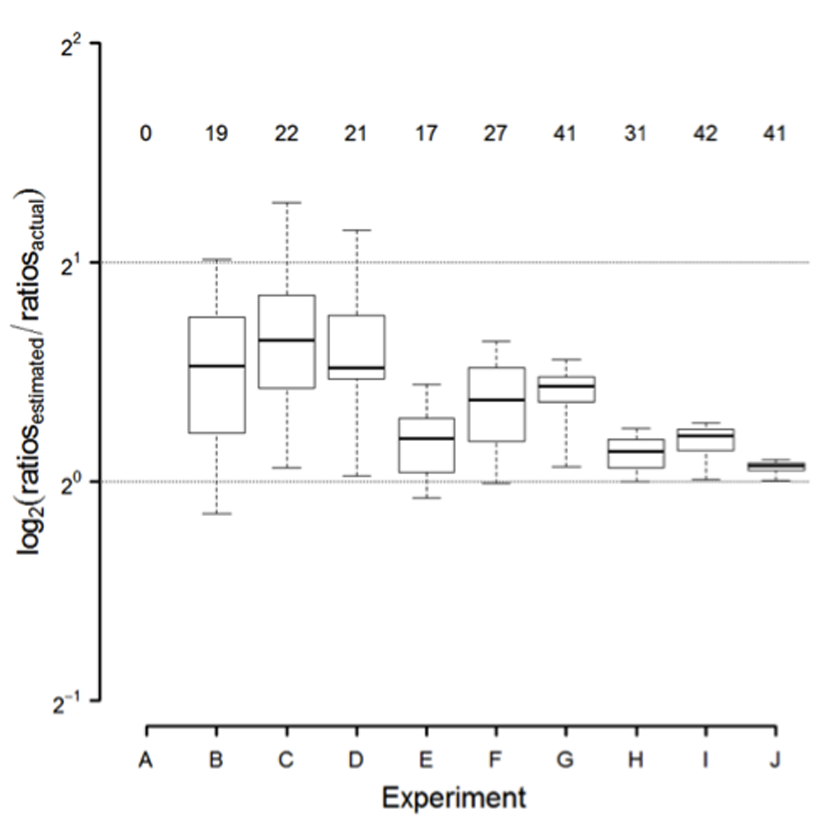

Supplement: S4 Fig — The analyses (experiment labels) are related to S3 Fig. Numbers inside the plot assign the number of proteins passing the significance thresholds. The accuracy increases with higher input ratios but not with increasing input amounts. Even small changes in a complex background proteome are elucidated. (TIF) [file pone.0159824.s004.tif]

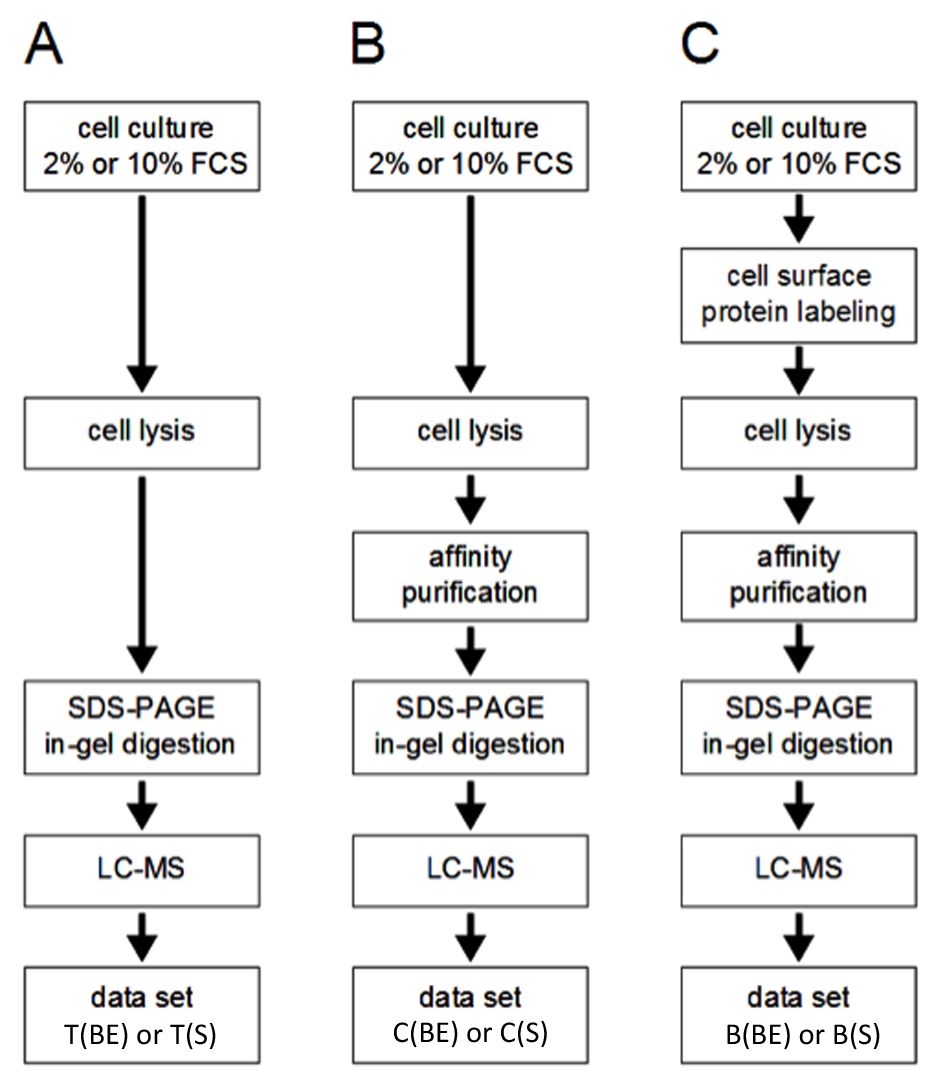

Supplement: S5 Fig — DPSCs were cultured either in basic expansion medium (2% FCS with PDGF and EGF additives) or in standard medium (10% FCS) (indices BE or S, respectively). A. Total proteome (samples T(BE) and T(S)). B. Control samples without labeling but with affinity purification (samples C(BE) and C(S)). C. Cell surface proteome-enrichment with biotinylation and affinity purification (samples B(BE) and B(S)). (TIF) [file pone.0159824.s005.tif]

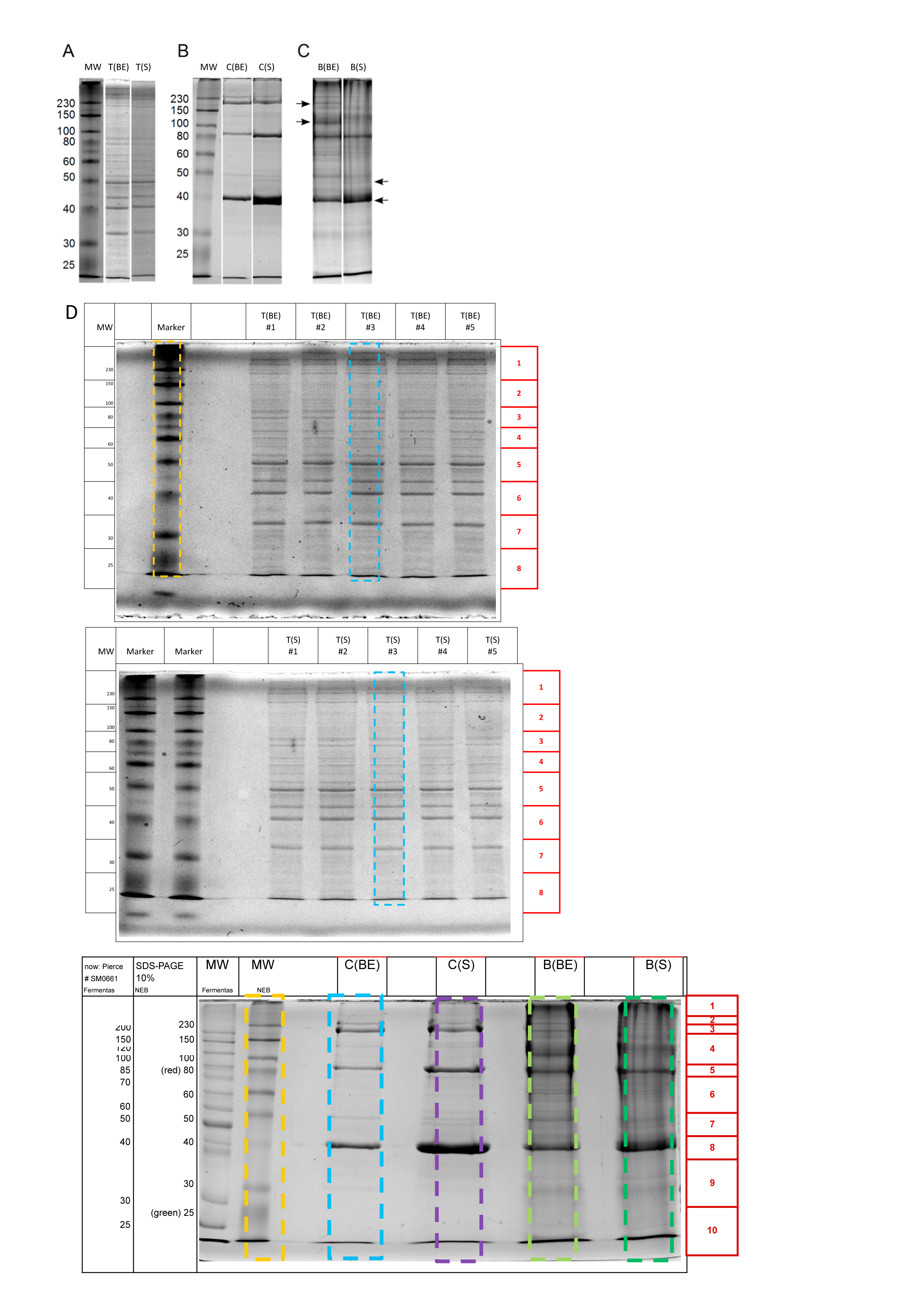

Supplement: S6 Fig — A. Gel lanes from the total proteome samples were indistinguishable in their band patterns. B. Gel lanes of the controls exhibited small differences. C. Arrows indicate some obvious differences in the gel band patterns of cell surface proteome-enriched samples. D. Uncropped gel images. Lanes used for A, B and C and cutting patterns are indicated. (TIF) [file pone.0159824.s006.tif]

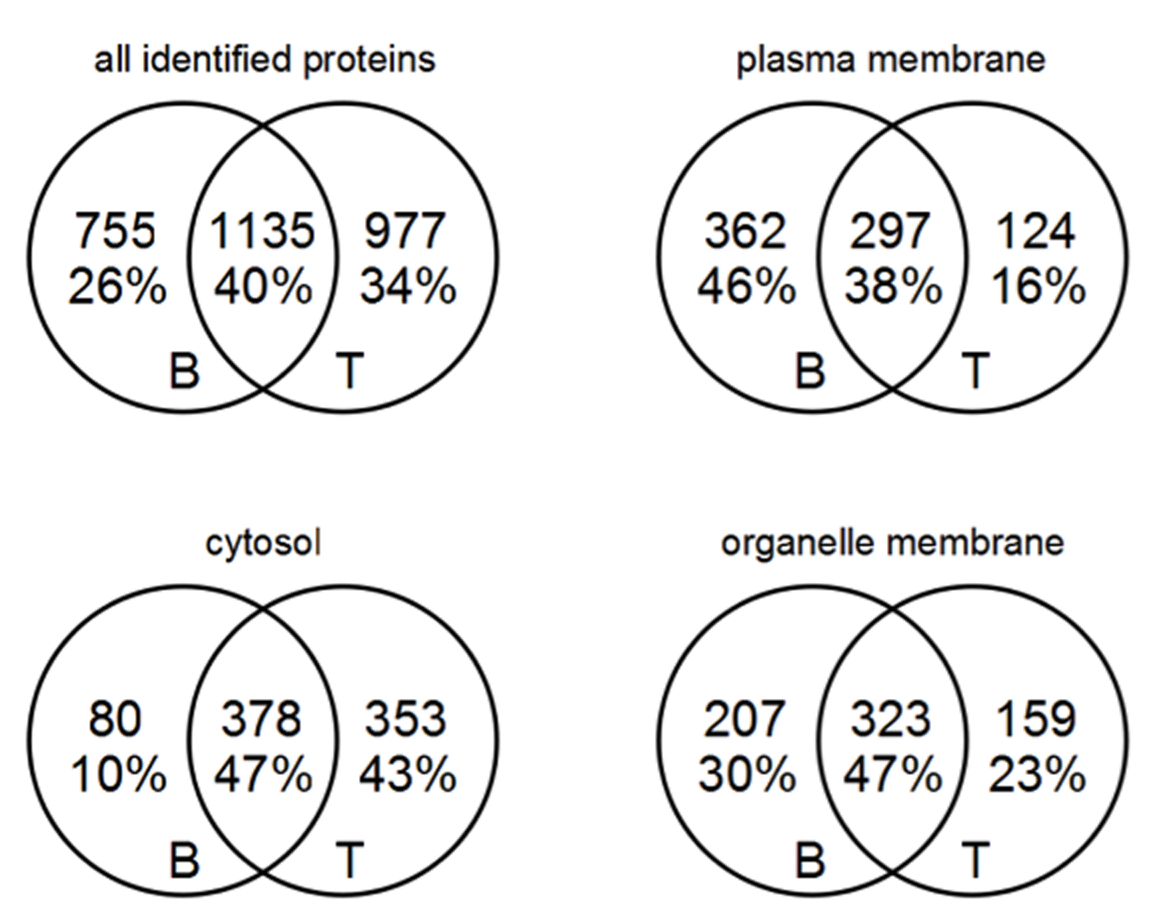

Supplement: S7 Fig — Venn-diagrams of cell surface proteome-enriched (B) and total proteome (T) samples. Around 40% of the 2,867 identified proteins were found in both types of samples. The cell surface proteome-enriched sample exhibited an approximately three fold higher number of proteins with a gene ontology assignment to plasma membrane localization (GO:0005886), whereas the total proteome sample contained four times more cytosol-localizing proteins (GO:0005829). Proteins assigned to organelle membranes (GO:0031090) were not preferentially enriched in either of the samples. (TIF) [file pone.0159824.s007.tif]

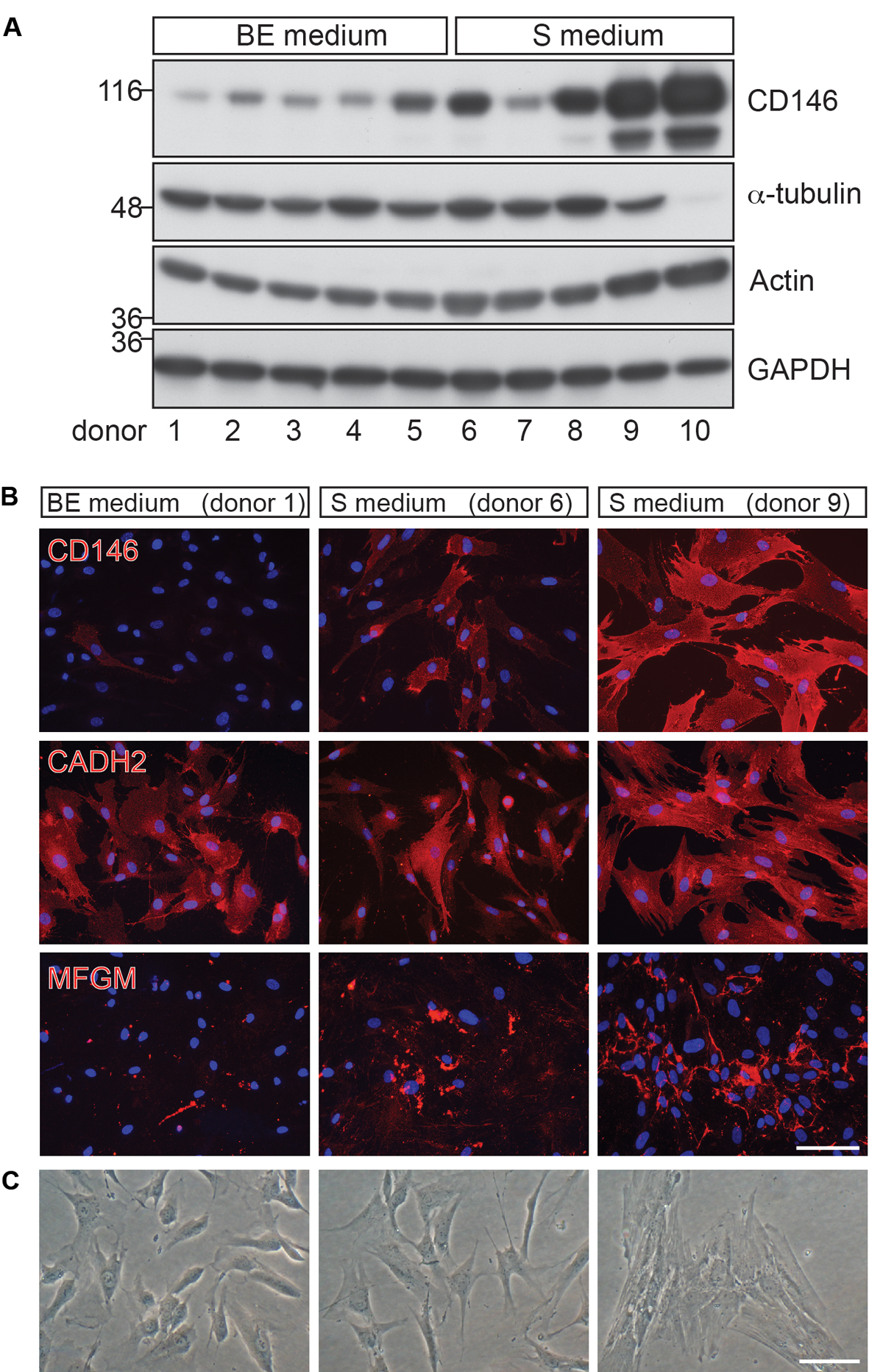

Supplement: S8 Fig — A. The expression profile of proteins acquired from DPSCs of 10 independent donors and cultured either in basic expansion (BE) or standard (S) media as indicated was examined by immunoblotting. Glyceraldehyde 3-phosphate dehydrogenase (GAPDH) was used as loading control. The position of prestained molecular mass markers is indicated on the left. In addition to CD146, note the variation in actin and α-tubulin among donors (e.g. donors 9 and 10 by comparison to others) suggesting indirectly an alteration in the cellular morphology. B and C. DPSCs from donors 1, 6 and 9 were either cell surface labeled (CD146, MFGM) or fixed, permeabilized and labeled (CADH2) prior analysis by (B) fluorescence microscopy or observed by (C) phase contrast microscopy. Nuclei were visualized with DAPI. Representative immunoblots and microscopy images from two to three independent experiments are displayed. Scale bars, 50 μm. (TIF) [file pone.0159824.s008.tif]
