# Supplementary material for: Cell Surface Proteome of Dental Pulp Stem Cells Identified by Label-Free Mass Spectrometry
Source: PLoS One. 2016 Aug 4;11(8):e0159824. doi: 10.1371/journal.pone.0159824 (PMC4973913; doi:10.1371/journal.pone.0159824)

Uncropped gels presented in Fig. 6A

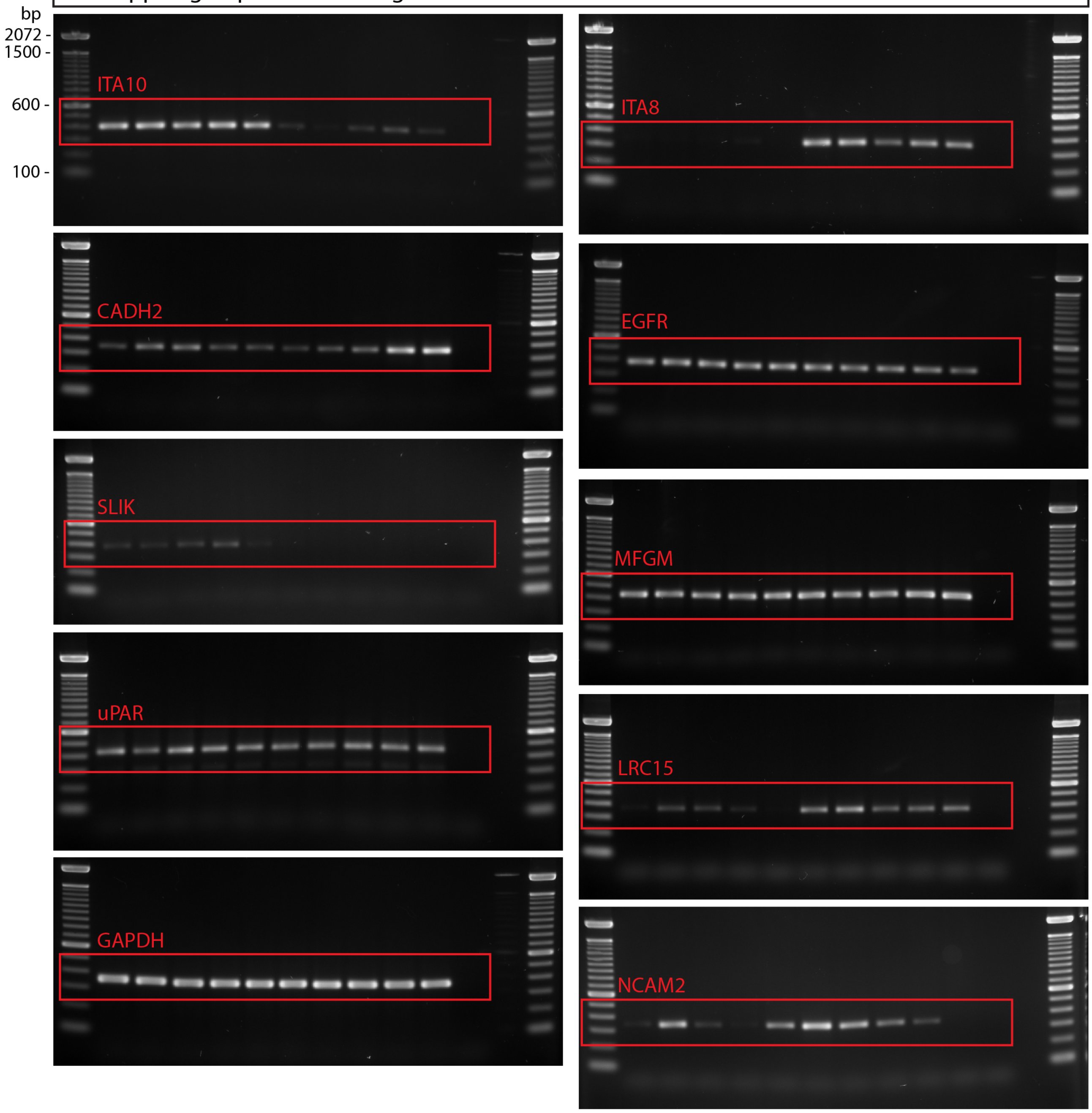

Uncropped immunoblots presented in Fig. 6C

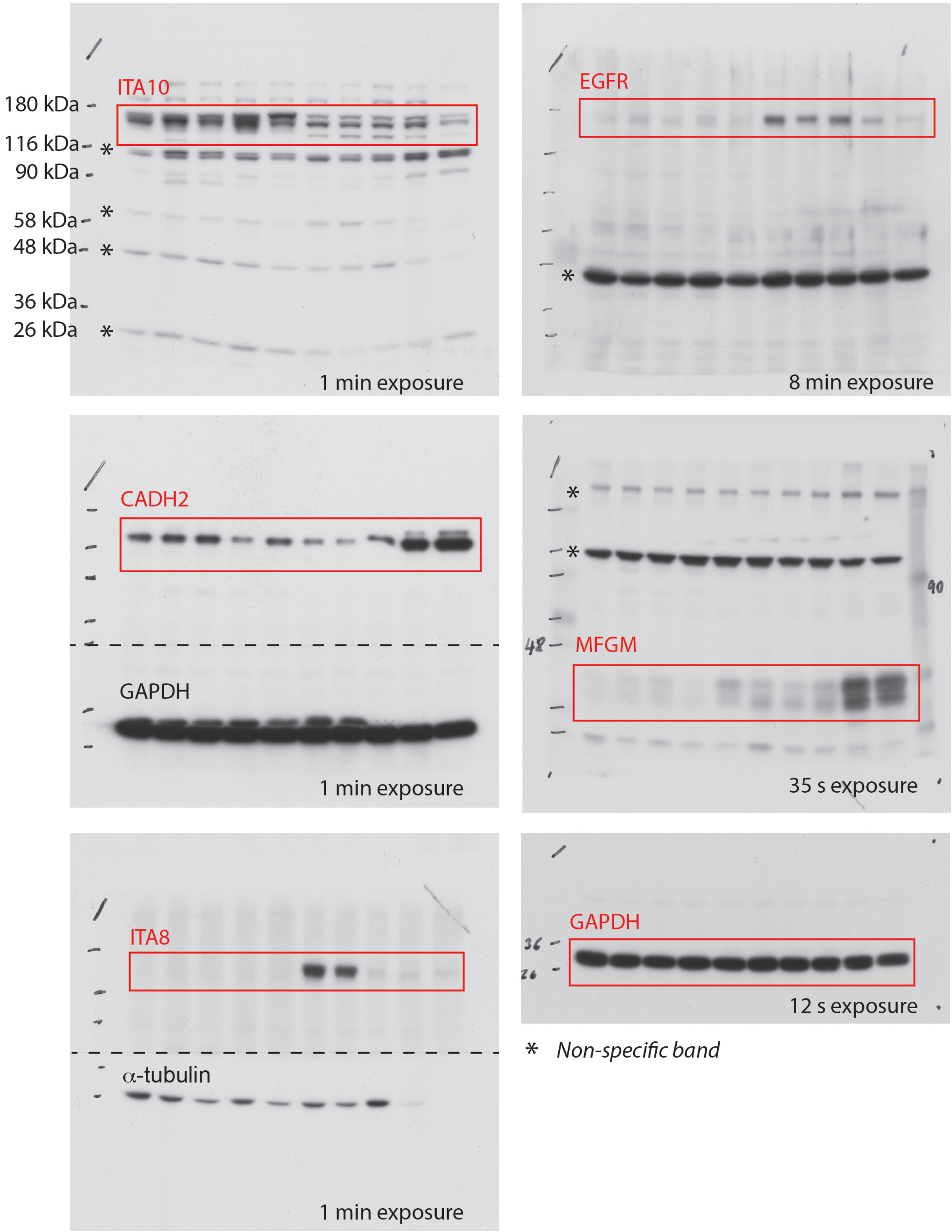

* *Non-specific band*

Uncropped immunoblots presented in Fig. S8A

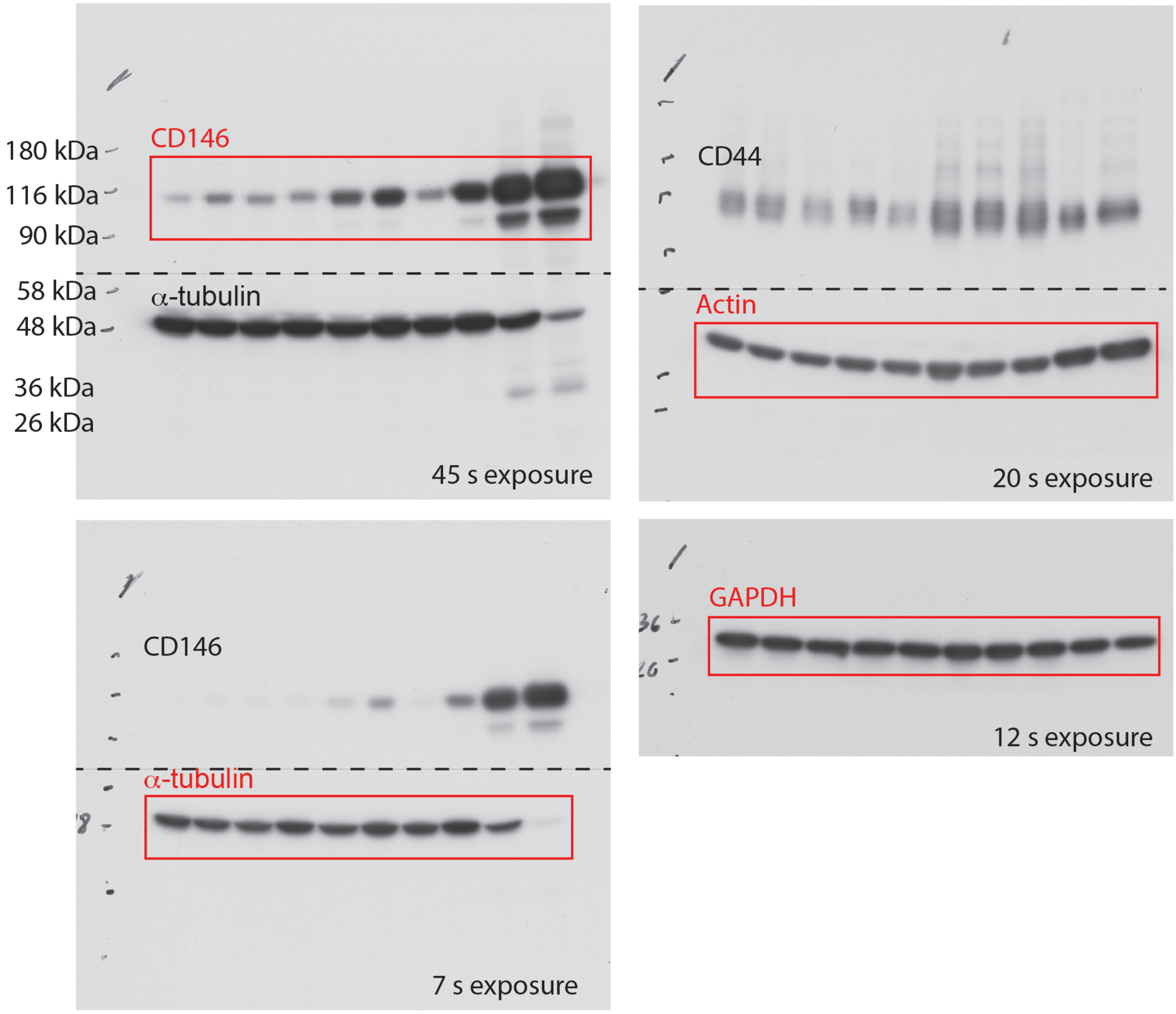

Supplement: S9 Fig — The relevant information presented in Fig 6A and 6C and S8A Fig are indicated in red boxes. DNA ladders and the molecular weight markers are presented on the left. Dashed line indicates the cut of PVDF membrane in 2 segments that were incubated with distinct primary antibodies. (PDF) [file pone.0159824.s009.pdf]
