# Supplementary material for: Cell Surface Proteome of Dental Pulp Stem Cells Identified by Label-Free Mass Spectrometry
Source: PLoS One. 2016 Aug 4;11(8):e0159824. doi: 10.1371/journal.pone.0159824 (PMC4973913; doi:10.1371/journal.pone.0159824)
Supplement: S1 Method — (DOCX) [file pone.0159824.s010.docx]

S1 Method

Python-script to facilitate ascertaining of retention time limits.

import csv

from pyteomics import fasta, parser, mass, achrom, electrochem, auxiliary

def run():

ini = iniFile("RCLimits.ini")

writer=csv.writer(open(ini.getSetting('OutPut','RCLimits.csv'), 'wb'), delimiter='\t',

quotechar='|', quoting=csv.QUOTE_MINIMAL)

reader=csv.reader(open(ini.getSetting('FileName','peptides.csv'), 'rb'), delimiter='\t',

quotechar='|')

firstRow = True

seqColumn = 0

for row in reader:

if firstRow:

firstRow = False

header = []

for entryNum in range(len(row)):

header.append(row[entryNum])

if row[entryNum]=='Sequence':

seqColumn = entryNum

header.append('RT_RP')

header.append('RT_normal')

writer.writerow(header)

else:

try:

rowToWrite = []

for j in range(seqColumn+1):

rowToWrite.append(row[j])

parsed = parser.parse( row[seqColumn], show_unmodified_termini=True)

RT_RP = achrom.calculate_RT(parsed, achrom.RCs_zubarev)

RT_normal = achrom.calculate_RT(parsed, achrom.RCs_yoshida_lc)

rowToWrite.append(str(RT_RP))

rowToWrite.append(str(RT_normal))

for i in range(len(row)-1-seqColumn):

rowToWrite.append(row[seqColumn+1+i])

writer.writerow(rowToWrite)

except Exception:

print "Errored Sequence"

class iniFile(object):

def __init__(self, fname):

self.fname = fname

self.settings = {}

self.read()

def printOut(self):

for setting in self.settings:

print setting + " : " + self.settings[setting]

def read(self):

try:

reader = csv.reader(open(self.fname, 'rb'), delimiter='=', quotechar='|')

for row in reader:

self.settings[row[0]]=row[1]

except Exception:

print "An error occured while reading "+self.fname

def getSetting(self, setting, default=""):

try:

return self.settings[setting]

except Exception:

return default

def getSettingInt(self, setting, default=0):

try:

return int(self.settings[setting])

except Exception:

return default

def getSettingFloat(self, setting, default=0.0):

try:

return float(self.settings[setting])

except Exception:

return default

def __del__(self):

del self.fname

del self.settings

if (__name__=="__main__"):

run()
