# Supplementary material for: Cell Surface Proteome of Dental Pulp Stem Cells Identified by Label-Free Mass Spectrometry
Source: PLoS One. 2016 Aug 4;11(8):e0159824. doi: 10.1371/journal.pone.0159824 (PMC4973913; doi:10.1371/journal.pone.0159824)
Supplement: S2 Method — (DOCX) [file pone.0159824.s011.docx]

**S2 Method**

Python-script to estimate numbers of observable tryptic peptides.

import csv

from pyteomics import fasta, parser, mass, achrom, electrochem, auxiliary

import re

from collections import deque

from itertools import chain, product

def run():

ini = iniFile('settings.ini')

debug = True

writeName = ini.getSetting('OutputPeptidesFileName', 'peptides.csv')

writeNameProt = ini.getSetting('OutputProteinsFileName', 'proteins.csv')

writingPeptides = True

if writeName == "false":

writingPeptides = False

writingProteins = True

if writeNameProt == "false":

writingProteins = False

errorWriter = csv.writer(open(ini.getSetting('ErrorLog','error.csv'), 'wb'),

delimiter='\t', quotechar='|', quoting=csv.QUOTE_MINIMAL)

csv.field_size_limit(1000000000)

if writingPeptides:

writer = csv.writer(open(writeName, 'wb'), delimiter='\t', quotechar='|',

quoting=csv.QUOTE_MINIMAL)

if writingProteins:

protWriter = csv.writer(open(writeNameProt, 'wb'), delimiter='\t', quotechar='|',

quoting=csv.QUOTE_MINIMAL)

if writingPeptides:

writer.writerow(['AccNo', 'PeptideSeq', 'RCrp', 'RCnorm', 'm', 'z', 'mz'])

if writingProteins:

protWriter.writerow(['AccNo', 'AccStr', 'GN', 'AA', 'm', 'NoP'])

try:

AAFilter = False

if(ini.getSetting('AA', 'false')=="true"):

print "AA-Filter enabled"

AAFilter = True

else:

print "AA-Filter disabled"

RTFilter = False

if(ini.getSetting('RT_RP', 'false')=="true"):

print "RT_RP-Filter enabled"

RTFilter = True

else:

print "RT_RP-Filter disabled"

RTNormFilter = False

if(ini.getSetting('RT_Norm', 'false')=="true"):

print "RT_normal-Filter enabled"

RTNormFilter = True

else:

print "RT_normal-Filter disabled"

ZFilter = False

if(ini.getSetting('Z', 'false')=="true"):

print "Z-Filter enabled"

ZFilter = True

else:

print "Z-Filter disabled"

pHValue = ini.getSettingFloat('pH')

proteinCount = 0

erroredPeptideCount = 0

for description, sequence in fasta.read(ini.getSetting('FileName', 'example.fasta')):

proteinCount += 1

new_peptides = cleave(sequence, parser.expasy_rules['trypsin'])

valid_peptides = []

AccNo = re.split('\|', description)[1]

AccStr = re.split('_', re.split('\|', description)[2])[0]

GN=""

if 'GN=' in re.split('\|', description)[2]:

for arg in re.split(' ', re.split('\|', description)[2]):

if arg.startswith('GN='):

GN=arg[3:]

ProtMass = ""

NoP = 0

erroredPeptideCountProt = 0

for p in new_peptides:

validPeptide = True

if AAFilter:

if len(str(p)) < ini.getSettingInt('AA-min'):

validPeptide = False

elif len(str(p)) > ini.getSettingInt('AA-max'):

validPeptide = False

if validPeptide:

valid_peptides.append(p)

new_peptides_length = len(new_peptides)

del new_peptides

peptides = [{'sequence':i} for i in valid_peptides]

del valid_peptides

for peptide in peptides:

try:

peptide['parsed_sequence'] = parser.parse( peptide['sequence'],

show_unmodified_termini=True)

peptide['valid'] = True

except Exception:

errorWriter.writerow([AccNo, peptide['sequence']])

peptide['valid'] = False

if peptide['valid']:

peptide['length'] = parser.length(peptide['parsed_sequence'])

for peptide in peptides:

if peptide['valid']:

peptide['charge'] = int(round(electrochem.charge(

peptide['parsed_sequence'], pH=pHValue)))

peptide['mass'] = mass.calculate_mass(peptide['parsed_sequence'])

peptide['m/z'] = mass.calculate_mass(

peptide['parsed_sequence'], charge=peptide['charge'])

for peptide in peptides:

if peptide['valid']:

peptide['RT_RP'] = achrom.calculate_RT(peptide['parsed_sequence'],

achrom.RCs_zubarev)

peptide['RT_normal'] = achrom.calculate_RT(peptide['parsed_sequence'],

achrom.RCs_yoshida_lc)

counter = 0

while counter < len(peptides):

peptide = peptides[counter]

removed = False

if peptide['valid']:

if RTFilter:

if peptide['RT_RP'] < ini.getSettingInt('RT_RP-min'):

peptides.remove(peptide)

removed = True

elif peptide['RT_RP'] > ini.getSettingInt('RT_RP-max'):

peptides.remove(peptide)

removed = True

if RTNormFilter and not removed:

if peptide['RT_normal'] < ini.getSettingInt('RT_Norm-min'):

peptides.remove(peptide)

removed = True

elif peptide['RT_normal'] > ini.getSettingInt('RT_Norm-max'):

peptides.remove(peptide)

removed = True

if ZFilter and not removed:

if peptide['charge'] < ini.getSettingInt('Z-min'):

peptides.remove(peptide)

removed = True

elif peptide['charge'] > ini.getSettingInt('Z-max'):

peptides.remove(peptide)

removed = True

if not removed:

counter += 1

for peptide in peptides:

try:

rp = peptide['RT_RP']

if writingPeptides:

writer.writerow([AccNo, peptide['sequence'], peptide['RT_RP'],

peptide['RT_normal'], peptide['mass'], peptide['charge'],

peptide['m/z']])

except Exception:

erroredPeptideCount += 1

erroredPeptideCountProt += 1

NoP = len(peptides)-erroredPeptideCountProt

try:

parsedProtein = parser.parse( sequence, show_unmodified_termini=True)

ProtMass = str(mass.calculate_mass(parsedProtein))

except Exception:

ProtMass = ""

if writingProteins:

protWriter.writerow([AccNo, AccStr, GN, str(len(sequence)), ProtMass,

str(NoP)])

print str(proteinCount) + " proteins parsed "

del peptides

del AccNo

except Exception:

print "An error occured while reading "+ini.getSetting('FileName', 'example.fasta')

del errorWriter

if writingPeptides:

del writer

if writingProteins:

del protWriter

print 'Done! '+str(erroredPeptideCount)+' peptides have failed to parse'

raw_input('')

del ini

def cleave(sequence, rule, missed_cleavages=0):

peptides = []

cleavage_sites = deque([0], maxlen=missed_cleavages+2)

for i in chain(map(lambda x: x.end(), re.finditer(rule, sequence)),

[None]):

cleavage_sites.append(i)

for j in range(0, len(cleavage_sites)-1):

peptides.append(sequence[cleavage_sites[j]:cleavage_sites[-1]])

if '' in peptides:

peptides.remove('')

return peptides

class iniFile(object):

def __init__(self, fname):

self.fname = fname

self.settings = {}

self.read()

def printOut(self):

for setting in self.settings:

print setting + " : " + self.settings[setting]

def read(self):

try:

reader = csv.reader(open(self.fname, 'rb'), delimiter='=', quotechar='|')

for row in reader:

self.settings[row[0]]=row[1]

except Exception:

print "An error occured while reading "+self.fname

def getSetting(self, setting, default=""):

try:

return self.settings[setting]

except Exception:

return default

def getSettingInt(self, setting, default=0):

try:

return int(self.settings[setting])

except Exception:

return default

def getSettingFloat(self, setting, default=0.0):

try:

return float(self.settings[setting])

except Exception:

return default

def __del__(self):

del self.fname

del self.settings

if(__name__=="__main__"):

run()
