# Supplementary material for: Cell Surface Proteome of Dental Pulp Stem Cells Identified by Label-Free Mass Spectrometry
Source: PLoS One. 2016 Aug 4;11(8):e0159824. doi: 10.1371/journal.pone.0159824 (PMC4973913; doi:10.1371/journal.pone.0159824)
Supplement: S3 Method — (DOCX) [file pone.0159824.s012.docx]

**S3 Method**

Parameter-file for listing I (RCLimits.ini).

FileName=evidence.txt

OutPut=evidence_out.txt
