# Supplementary material for: Cell Surface Proteome of Dental Pulp Stem Cells Identified by Label-Free Mass Spectrometry
Source: PLoS One. 2016 Aug 4;11(8):e0159824. doi: 10.1371/journal.pone.0159824 (PMC4973913; doi:10.1371/journal.pone.0159824)
Supplement: S4 Method — (DOCX) [file pone.0159824.s013.docx]

**S4 Method**

Parameter-file for listing II (Settings.ini).

AA=true

AA-min=6

AA-max=60

RT_RP=false

RT_RP-min=5

RT_RP-max=35

Z=true

Z-min=2

Z-max=3

RT_Norm=false

RT_Norm-min=0

RT_Norm-max=0

Gradient_Length=40

pH=3.0

FileName=human.fasta

OutputPeptidesFileName=peptides.csv

OutputProteinsFileName=proteins.csv

Path=C:\Users\Admin\Python

ErrorLog=error.csv
