# Supplementary material for: Cell Surface Proteome of Dental Pulp Stem Cells Identified by Label-Free Mass Spectrometry
Source: PLoS One. 2016 Aug 4;11(8):e0159824. doi: 10.1371/journal.pone.0159824 (PMC4973913; doi:10.1371/journal.pone.0159824)
Supplement: S5 Method — (DOCX) [file pone.0159824.s014.docx]

**S5 Method**

Boolean expression to create a list of and to filter for cell surface proteins

organism:homo sapiens AND reviewed:yes AND (( GO:0005886 AND (keyword:GPI-anchor OR annotation:(type:transmem count:[1 TO *])) ) OR ((GO:0005615 OR GO:0005576 OR GO:0031012) AND annotation:(type:signal peptide)))

4508 matches in Uniprot (Version 2011_01). GO:0005886 - plasma membrane – PM, GO:0005615 - extracellular space – ECS, GO:0005576 - extracellular region – ECR, GO:0031012 - extracellular matrix - ECM
