# Supplementary material for: Cell Surface Proteome of Dental Pulp Stem Cells Identified by Label-Free Mass Spectrometry
Source: PLoS One. 2016 Aug 4;11(8):e0159824. doi: 10.1371/journal.pone.0159824 (PMC4973913; doi:10.1371/journal.pone.0159824)
Supplement: S1 Table — (DOCX) [file pone.0159824.s015.docx]

**S1 Table.** List of primary antibodies used for immunodetection

| **Antigen** | **Clone** | **Manufacturer** | **Dilution** | | |
| --- | --- | --- | --- | --- | --- |
|  |  |  | **WB** | **FC** | **ICC** |
| Actin | AC-40 | Sigma-Aldrich | 1:5000 |  |  |
| α-tubulin | YOL-1/34 | Serotec | 1:500 |  |  |
| CADH2 | 32/N-cadherin | BD Biosciences | 1:1000 |  | 1:50 |
| CD9 | M-L13 | BD Biosciences |  |  | 1:50 |
| CD9-PE | HI9a | BioLegend |  | 1:10 |  |
| CD39 | A1 | Invitrogen |  | 1:20 | 1:50 |
| CD44 | DF1485 | Santa Cruz Biotechn. |  |  | 1:50 |
| CD44-PE | G44-26 | BD Biosciences |  | 1:20 |  |
| CD106 | 51-10C9 | BD Biosciences |  | 1:20 | 1:50 |
| CD146 | P1H12 | BD Biosciences |  |  | 1:50 |
| CD146 | N1238 | Leica Biosystems | 1:250 |  |  |
| CD151 | 11G5a | Acris Antibodies |  | 1:100 | 1:100 |
| CD166 | 3A6 | BD Biosciences |  |  | 1:50 |
| CD166-PE | 3A6 | BD Biosciences |  | 1:5 |  |
| EGFR | antiserum^1^ | Sigma-Aldrich | 1:1000 |  |  |
| GAPDH | 6C5 | Acris Antibodies | 1:5000 |  |  |
| ITA8 | 481709 | R&D systems | 1:1000 |  |  |
| ITA10 | antiserum^1^ | Millipore | 1:2500 | 1:100 | 1:100 |
| MFGM (MFG-E8) | 278901 | R&D systems | 1:300 | 1:20 | 1:50 |
| MFGM (MFG-E8) | antiserum^1^ | Santa Cruz Biotechn. | 1:200 |  |  |
| uPAR-PE (CD87) | VIM5 | BioLegend |  | 1:5 | 1:20 |

^1^Antibody generated in rabbit.

FC, flow cytometry; ICC, immunocytochemistry; PE, phycoerythrin; WB, immunoblotting.
