# Supplementary material for: Cell Surface Proteome of Dental Pulp Stem Cells Identified by Label-Free Mass Spectrometry
Source: PLoS One. 2016 Aug 4;11(8):e0159824. doi: 10.1371/journal.pone.0159824 (PMC4973913; doi:10.1371/journal.pone.0159824)
Supplement: S2 Table — (DOCX) [file pone.0159824.s016.docx]

**S2 Table.** List of primers used for RT-PCR analysis

| **Gene** | **Accession number** | **Forward primer (5'->3')**  **Reverse primer (5'->3')** | **Product size**  **(bp)** |
| --- | --- | --- | --- |
| CADH2 | NM_001792 | CAATGCCCCTCAAGTGTTACC | 352 |
|  |  | ACCCACAATCCTGTCCACATC |  |
| EGFR | NM_005228 | TCGATGGACTTCCAGAACCAC | 387 |
|  |  | GCCGTGATCTGTCACCACATA |  |
| GAPDH | NM_002046 | CGGGAAGCTTGTCATCAATGG | 358 |
|  |  | GGCAGTGATGGCATGGACTG |  |
| ITA8 | NM_003638 | TGGACTTCCACATACCCGAC | 340 |
|  |  | GCTACATAGCAGGTGCCAAC |  |
| ITA10 | NM_003637 | CTTCCAAGTGACCTCCCGTA | 417 |
|  |  | CCACCTCGAACCACAAATGG |  |
| LRC15 | NM_001135057 | GTGTTTCAGCCCAGCCAATG | 368 |
|  |  | TCTGCATCAGGACAGCTTGG |  |
| MFGM | NM_001114614 | TGGCCAGTCATGAGTACCTG | 425 |
|  |  | CTGCAGCCACTGATCGTTAC |  |
| NCAM2 | NM_004540 | GTGAACCAAGTCCTCCATCCA | 347 |
|  |  | CAGGCCAATTACTGCTCCAAG |  |
| SLIK2 | NM_032539 | GTGACTTGCGAATCTCCTGCT | 382 |
|  |  | GGCCGTCTGTTTTATCGTGAG |  |
| uPAR | NM_002659 | TGAGCTATCGGACTGGCTTGA | 436 |
|  |  | TTCCCCTTGCAGCTGTAACAC |  |

Bp: Base pairs.
