# Supplementary material for: Cell Surface Proteome of Dental Pulp Stem Cells Identified by Label-Free Mass Spectrometry
Source: PLoS One. 2016 Aug 4;11(8):e0159824. doi: 10.1371/journal.pone.0159824 (PMC4973913; doi:10.1371/journal.pone.0159824)
Supplement: S3 Table — (DOCX) [file pone.0159824.s017.docx]

**S3 Table:** CD-marker proteins identified.

| **CD** | **Acc. No .** | **B** | **T** | **Name** |
| --- | --- | --- | --- | --- |
| CD9 | P21926 | ++++ | +++ | CD9 antigen |
| CD10 | P08473 | ++++ | +++ | Neprilysin |
| CD13 | P15144 | ++++ | ++++ | Aminopeptidase N |
| CD14 | P08571 | ++ | - | Monocyte differentiation antigen CD14 |
| CD29 | P05556 | ++++ | ++++ | Integrin beta-1 |
| CD39 | P49961 | ++ | - | Ectonucleoside triphosphate diphosphohydrolase 1 |
| CD40 | P25942 | +++ | - | Tumor necrosis factor receptor superfamily member 5 |
| CD44 | P16070 | ++++ | ++++ | CD44 antigen |
| CD46 | P15529 | +++ | - | Membrane cofactor protein |
| CD47 | Q08722 | ++++ | ++ | Leukocyte surface antigen CD47 |
| CD49a | P56199 | ++++ | - | Integrin alpha-1 |
| CD49b | P17301 | ++++ | ++++ | Integrin alpha-2 |
| CD49c | P26006 | ++++ | +++ | Integrin alpha-3 |
| CD49d | P13612 | ++++ | - | Integrin alpha-4 |
| CD49e | P08648 | ++++ | +++ | Integrin alpha-5 |
| CD49f | P23229 | +++ | - | Integrin alpha-6 |
| CD51 | P06756 | ++++ | +++ | Integrin alpha-V |
| CD54 | P05362 | ++ | - | Intercellular adhesion molecule 1 |
| CD55 | P08174 | +++ | - | Complement decay-accelerating factor |
| CD56 | P13591 | ++ | - | Neural cell adhesion molecule 1 |
| CD58 | P19256 | ++ | - | Lymphocyte function-associated antigen 3 |
| CD59 | P13987 | ++++ | +++ | CD59 glycoprotein |
| CD61 | P05106 | +++ | - | Integrin beta-3 |
| CD63 | P08962 | +++ | ++++ | CD63 antigen |
| CD68 | P34810 | ++ | - | Macrosialin |
| CD71 | P02786 | ++++ | ++++ | Transferrin receptor protein 1 |
| CD73 | P21589 | ++++ | ++++ | 5-nucleotidase |
| CD81 | P60033 | ++++ | +++ | CD81 antigen |
| CD82 | P27701 | ++ | - | CD82 antigen |
| CD87 | Q03405 | ++ | - | Urokinase plasminogen activator surface receptor |
| CD88 | P21730 | ++ | - | C5a anaphylatoxin chemotactic receptor |
| CD90 | P04216 | ++++ | +++ | Thy-1 membrane glycoprotein |
| CD91 | Q07954 | ++++ | ++++ | Prolow-density lipoprotein receptor-related protein 1 |
| CD92 | Q8WWI5 | ++++ | ++ | Choline transporter-like protein 1 |
| CD95 | P25445 | +++ | - | Tumor necrosis factor receptor superfamily member 6 |
| CD97 | P48960 | ++++ | - | CD97 antigen |
| CD98 | P08195 | ++++ | +++ | 4F2 cell-surface antigen heavy chain |
| CD99 | P14209 | +++ | - | CD99 antigen |
| CD105 | P17813 | ++++ | - | Endoglin |
| CD106 | P19320 | ++ | - | Vascular cell adhesion protein 1 |
| CD107a | P11279 | +++ | ++++ | Lysosome-associated membrane glycoprotein 1 |
| CD107b | P13473 | - | +++ | Lysosome-associated membrane glycoprotein 2 |
| CD108 | O75326 | ++ | ++ | Semaphorin-7A |
| CD109 | Q6YHK3 | ++++ | ++++ | CD109 antigen |
| CD112 | Q92692 | ++++ | - | Poliovirus receptor-related protein 2 |
| CD113 | Q9NQS3 | ++++ | - | Poliovirus receptor-related protein 3 |
| CD119 | P15260 | ++ | - | Interferon gamma receptor 1 |
| CD120a | P19438 | ++ | - | Tumor necrosis factor receptor superfamily member 1A |
| CD121a | P14778 | ++ | - | Interleukin-1 receptor type 1 |
| CD130 | P40189 | +++ | - | Interleukin-6 receptor subunit beta |
| CD140a | P16234 | ++++ | ++ | Alpha-type platelet-derived growth factor receptor |
| CD140b | P09619 | ++++ | +++ | Beta-type platelet-derived growth factor receptor |
| CD141 | P07204 | ++ | - | Thrombomodulin |
| CD142 | P13726 | ++ | - | Tissue factor |
| CD143 | P12821 | ++++ | - | Angiotensin-converting enzyme |
| CD146 | P43121 | +++ | - | Cell surface glycoprotein MUC18 |
| CD147 | P35613 | ++++ | +++ | Basigin |
| CD148 | Q12913 | ++++ | - | Receptor-type tyrosine-protein phosphatase eta |
| CD151 | P48509 | ++++ | +++ | CD151 antigen |
| CD155 | P15151 | ++++ | - | Poliovirus receptor |
| CD156b | P78536 | +++ | - | Disintegrin and metalloproteinase domain-containing protein 17 |
| CD156c | O14672 | ++++ | ++ | Disintegrin and metalloproteinase domain-containing protein 10 |
| CD157 | Q10588 | ++ | - | ADP-ribosyl cyclase 2 |
| CD166 | Q13740 | ++++ | ++++ | CD166 antigen |
| CD167b | Q16832 | ++++ | ++ | Discoidin domain-containing receptor 2 |
| CD172a | P78324 | +++ | - | Tyrosine-protein phosphatase non-receptor type substrate 1 |
| CD201 | Q9UNN8 | ++++ | - | Endothelial protein C receptor |
| CD203a | P22413 | ++++ | - | Ectonucleotide pyrophosphatase/phosphodiesterase family member 1 |
| CD213a1 | P78552 | + | - | Interleukin-13 receptor subunit alpha-1 |
| CD217 | Q96F46 | +++ | - | Interleukin-17 receptor A |
| CD221 | P08069 | +++ | - | Insulin-like growth factor 1 receptor |
| CD222 | P11717 | ++++ | +++ | Cation-independent mannose-6-phosphate receptor |
| CD224 | P19440 | ++ | - | Gamma-glutamyltranspeptidase 1 |
| CD230 | P04156 | ++ | - | Major prion protein |
| CD232 | O60486 | ++ | - | Plexin-C1 |
| CD236 | P04921 | +++ | - | Glycophorin-C |
| CD239 | P50895 | +++ | - | Basal cell adhesion molecule |
| CD248 | Q9HCU0 | ++++ | +++ | Endosialin |
| CD261 | O00220 | ++ | - | Tumor necrosis factor receptor superfamily member 10A |
| CD262 | O14763 | +++ | - | Tumor necrosis factor receptor superfamily member 10B |
| CD264 | Q9UBN6 | ++ | - | Tumor necrosis factor receptor superfamily member 10D |
| CD266 | Q9NP84 | ++ | - | Tumor necrosis factor receptor superfamily member 12A |
| CD273 | Q9BQ51 | +++ | - | Programmed cell death 1 ligand 2 |
| CD274 | Q9NZQ7 | ++ | - | Programmed cell death 1 ligand 1 |
| CD276 | Q5ZPR3 | ++++ | ++ | CD276 antigen |
| CD277 | O00481 | ++ | - | Butyrophilin subfamily 3 member A1 |
| CD280 | Q9UBG0 | ++++ | +++ | C-type mannose receptor 2 |
| CD282 | O60603 | + | - | Toll-like receptor 2 |
| CD292 | P36894 | ++ | - | Bone morphogenetic protein receptor type-1A |
| CD295 | P48357 | +++ | - | Leptin receptor |
| CD298 | P54709 | ++++ | +++ | Sodium/potassium-transporting ATPase subunit beta-3 |
| CD302 | Q8IX05 | ++ | - | CD302 antigen |
| CD304 | O14786 | ++++ | +++ | Neuropilin-1 |
| CD316 | Q969P0 | ++++ | - | Immunoglobulin superfamily member 8 |
| CD318 | Q9H5V8 | +++ | - | CUB domain-containing protein 1 |
| CD325 | P19022 | +++ | - | Cadherin-2 |
| CD331 | P11362 | +++ | - | Basic fibroblast growth factor receptor 1 |
| CD339 | P78504 | ++ | - | Protein jagged-1 |
| CD340 | P04626 | +++ | - | Receptor tyrosine-protein kinase erbB-2 |
| CD362 | P34741 | ++ | - | Syndecan-2 |
| CDw210b | Q08334 | ++ | - | Interleukin-10 receptor subunit beta |

CD-marker proteins not identified: CD1a, CD1b, CD1c, CD1d, CD1e, CD2, CD3d, CD3e, CD3g, CD4, CD5, CD6, CD7, CD8a, CD8b, CD11a, CD11b, CD11c, CD11d, CD16a, CD16b, CD18, CD19, CD20, CD21, CD22, CD23, CD24, CD25, CD26, CD27, CD28, CD30, CD31, CD32, CD33, CD34, CD35, CD36, CD37, CD38, CD41, CD42a, CD42b, CD42c, CD42d, CD43, CD45, CD48, CD50, CD52, CD53, CD62E, CD62L, CD62P, CD64, CD66a, CD66b, CD66c, CD66d, CD66e, CD66f, CD69, CD70, CD72, CD74, CD79a, CD79b, CD80, CD83, CD84, CD85a, CD85c, CD85d, CD85e, CD85f, CD85g, CD85h, CD85i, CD85j, CD85k, CD86, CD89, CD93, CD94, CD96, CD100, CD101, CD102, CD103, CD104, CD110, CD111, CD114, CD115, CD116, CD117, CD118, CD120b, CD121b, CD122, CD123, CD124, CD125, CD126, CD127, CD129, CD131, CD132, CD133, CD134, CD135, CD136, CD137, CD138, CD144, CD150, CD152, CD153, CD154, CD156a, CD158a, CD158b1, CD158b2, CD158c, CD158d, CD158e, CD158f1, CD158f2, CD158g, CD158h, CD158i, CD158j, CD158k, CD158z, CD159a, CD159c, CD160, CD161, CD162, CD163, CD163b, CD164, CD167a, CD168, CD169, CD170, CD171, CD172b, CD172g, CD177, CD178, CD179a, CD179b, CD180, CD181, CD182, CD183, CD184, CD185, CD186, CD191, CD192, CD193, CD194, CD195, CD196, CD197, CDw198, CDw199, CD200, CD202b, CD203c, CD204, CD205, CD206, CD207, CD208, CD209, CD210, CD212, CD213a2, CD215, CD218a, CD218b, CD220, CD223, CD225, CD226, CD227, CD228, CD229, CD231, CD233, CD234, CD235a, CD235b, CD238, CD240CE, CD240D, CD241, CD242, CD243, CD244, CD246, CD247, CD249, CD252, CD253, CD254, CD256, CD257, CD258, CD263, CD265, CD267, CD268, CD269, CD270, CD271, CD272, CD275, CD278, CD279, CD281, CD283, CD284, CD286, CD288, CD289, CD290, CDw293, CD294, CD296, CD297, CD299, CD300a, CD300b, CD300c, CD300d, CD300e, CD300f, CD300g, CD301, CD303, CD305, CD306, CD307a, CD307b, CD307c, CD307d, CD307e, CD309, CD312, CD314, CD315, CD317, CD319, CD320, CD321, CD322, CD324, CD326, CD327, CD328, CD329, CD332, CD333, CD334, CD335, CD336, CD337, CD338, CD344, CD349, CD350, CD351, CD352, CD353, CD354, CD355, CD357, CD358, CD360, CD361, CD363
