# Supplementary material for: Cell Surface Proteome of Dental Pulp Stem Cells Identified by Label-Free Mass Spectrometry
Source: PLoS One. 2016 Aug 4;11(8):e0159824. doi: 10.1371/journal.pone.0159824 (PMC4973913; doi:10.1371/journal.pone.0159824)
Supplement: S4 Table — (DOCX) [file pone.0159824.s018.docx]

**S4 Table.** Non CD-marker cell surface proteins identified.

| **SP** | **TM** | **GA** | **PM** | **ECS** | **ECR** | **ECM** | **AccNo** | **B** | **T** | **Name** |
| --- | --- | --- | --- | --- | --- | --- | --- | --- | --- | --- |
|  | + |  | + |  |  |  | Q15125 | ++ | - | 3-beta-hydroxysteroid-Delta(8),Delta(7)-isomerase |
| + |  |  |  | + | + | + | P08253 | ++ | - | 72 kDa type IV collagenase |
| + | + |  | + |  |  |  | Q04771 | +++ | - | Activin receptor type-1 |
|  | + |  | + |  |  |  | P51828 | ++ | - | Adenylate cyclase type 7 |
|  | + |  | + |  |  |  | O60503 | +++ | - | Adenylate cyclase type 9 |
| + | + |  | + |  |  |  | Q9H6B4 | ++++ | - | Adipocyte adhesion molecule |
| + |  |  |  | + | + |  | Q8IUX7 | ++ | - | Adipocyte enhancer-binding protein 1 |
|  | + |  | + |  |  |  | P12235 | + | - | ADP/ATP translocase 1 |
|  | + |  | + |  |  |  | P05141 | +++ | +++ | ADP/ATP translocase 2 |
| + |  |  |  |  | + |  | Q9BRR6 | ++ | ++ | ADP-dependent glucokinase |
| + |  |  |  | + | + |  | P43652 | ++ | - | Afamin |
| + |  | + | + | + | + |  | P05186 | ++ | - | Alkaline phosphatase, tissue-nonspecific isozyme |
| + |  |  |  |  | + |  | P04217 | ++ | - | Alpha-1B-glycoprotein |
| + |  |  |  | + | + | + | P02765 | ++ | - | Alpha-2-HS-glycoprotein |
| + |  |  | + |  | + |  | P30533 | +++ | - | Alpha-2-macroglobulin receptor-associated protein |
| + | + |  | + |  |  |  | Q86SJ2 | ++ | - | Amphoterin-induced protein 2 |
| + |  |  |  | + | + |  | Q9UKU9 | ++ | - | Angiopoietin-related protein 2 |
|  | + |  | + |  |  |  | P04920 | ++ | - | Anion exchange protein 2 |
|  | + |  | + |  |  |  | Q9NW15 | - | +++ | Anoctamin-10 |
|  | + |  | + |  |  |  | Q4KMQ2 | ++++ | - | Anoctamin-6 |
| + | + |  | + |  |  |  | Q9H6X2 | +++ | - | Anthrax toxin receptor 1 |
| + | + |  | + |  | + |  | P58335 | +++ | - | Anthrax toxin receptor 2 |
|  | + |  | + |  |  |  | Q03518 | ++ | - | Antigen peptide transporter 1 |
|  | + |  | + |  |  |  | Q03519 | ++ | - | Antigen peptide transporter 2 |
| + |  |  | + | + | + |  | P04114 | ++ | - | Apolipoprotein B-100 |
|  | + |  | + |  |  |  | Q12797 | +++ | ++++ | Aspartyl/asparaginyl beta-hydroxylase |
|  | + |  | + |  |  |  | O95477 | +++ | - | ATP-binding cassette sub-family A member 1 |
|  | + |  | + |  |  |  | O94911 | ++ | - | ATP-binding cassette sub-family A member 8 |
| + | + |  | + |  |  |  | P20594 | +++ | - | Atrial natriuretic peptide receptor 2 |
| + | + |  | + |  |  |  | P17342 | ++ | - | Atrial natriuretic peptide receptor 3 |
|  | + |  | + |  | + |  | Q9Y5Q5 | ++ | - | Atrial natriuretic peptide-converting enzyme |
| + | + |  | + | + | + |  | O75882 | +++ | - | Attractin |
|  | + |  | + |  |  |  | P30411 | +++ | - | B2 bradykinin receptor |
| + |  |  | + | + | + | + | P98160 | - | +++ | Basement membrane-specific heparan sulfate proteoglycan core protein |
|  | + |  | + |  |  |  | P51572 | ++ | +++ | B-cell receptor-associated protein 31 |
|  | + |  | + |  |  |  | P07550 | ++ | - | Beta-2 adrenergic receptor |
| + |  |  | + | + | + |  | P61769 | +++ | - | Beta-2-microglobulin |
|  | + |  | + |  |  |  | Q16585 | +++ | - | Beta-sarcoglycan |
| + | + |  | + |  |  |  | Q13873 | +++ | - | Bone morphogenetic protein receptor type-2 |
| + | + |  | + |  |  |  | Q7KYR7 | +++ | - | Butyrophilin subfamily 2 member A1 |
|  | + |  | + |  |  |  | Q9P296 | ++ | - | C5a anaphylatoxin chemotactic receptor C5L2 |
| + | + |  | + |  |  |  | P55287 | ++++ | - | Cadherin-11 |
| + |  | + | + | + | + |  | P55290 | ++ | - | Cadherin-13 |
| + | + |  | + |  |  |  | P55283 | ++ | - | Cadherin-4 |
| + | + |  | + |  |  |  | P55285 | ++ | ++ | Cadherin-6 |
|  | + |  | + |  |  |  | Q96D31 | ++ | - | Calcium release-activated calcium channel protein 1 |
|  | + |  | + |  |  |  | Q12791 | +++ | - | Calcium-activated potassium channel subunit alpha-1 |
|  | + |  | + |  |  |  | Q9UJS0 | - | +++ | Calcium-binding mitochondrial carrier protein Aralar2 |
| + |  |  | + | + | + | + | P27797 | +++ | ++++ | Calreticulin |
| + |  |  |  |  | + |  | O43852 | +++ | ++++ | Calumenin |
|  | + |  | + |  |  |  | O15438 | ++ | - | Canalicular multispecific organic anion transporter 2 |
| + | + |  | + |  |  |  | O43570 | ++ | - | Carbonic anhydrase 12 |
| + |  | + | + |  |  |  | P14384 | +++ | - | Carboxypeptidase M |
| + |  |  |  |  | + | + | O75718 | - | +++ | Cartilage-associated protein |
|  | + |  | + |  |  |  | P21964 | +++ | +++ | Catechol O-methyltransferase |
| + |  |  | + | + | + |  | P07858 | ++ | +++ | Cathepsin B |
| + |  |  |  | + | + |  | P07339 | ++ | +++ | Cathepsin D |
| + |  |  |  | + | + |  | P43235 | ++ | - | Cathepsin K |
| + |  |  |  | + | + |  | Q9UBR2 | - | +++ | Cathepsin Z |
| + | + |  | + |  |  |  | P20645 | +++ | - | Cation-dependent mannose-6-phosphate receptor |
| + | + |  | + |  |  |  | Q8TCZ2 | +++ | - | CD99 antigen-like protein 2 |
|  | + |  | + |  |  |  | O14735 | ++ |  | CDP-diacylglycerol--inositol 3-phosphatidyltransferase |
| + | + |  | + |  |  |  | Q9BY67 | ++ | - | Cell adhesion molecule 1 |
|  | + |  | + |  |  |  | Q99788 | ++ | - | Chemokine-like receptor 1 |
|  | + |  | + |  |  |  | O00299 | ++ | ++++ | Chloride intracellular channel protein 1 |
|  | + |  | + |  |  |  | Q9Y696 | ++ | ++++ | Chloride intracellular channel protein 4 |
|  | + |  | + |  |  |  | Q8IWA5 | ++++ | ++ | Choline transporter-like protein 2 |
| + | + |  | + |  | + |  | Q6UVK1 | ++++ | ++++ | Chondroitin sulfate proteoglycan 4 |
|  | + |  | + |  |  |  | Q9NY35 | +++ | - | Claudin domain-containing protein 1 |
|  | + |  | + |  |  |  | O96005 | - | +++ | Cleft lip and palate transmembrane protein 1 |
| + |  |  |  | + | + | + | P02452 | +++ | ++++ | Collagen alpha-1(I) chain |
| + |  |  |  |  | + | + | P20908 | ++ | ++ | Collagen alpha-1(V) chain |
| + |  |  | + |  | + | + | P12109 | ++ | ++++ | Collagen alpha-1(VI) chain |
| + |  |  |  |  | + | + | Q02388 | - | ++ | Collagen alpha-1(VII) chain |
| + |  |  |  | + | + | + | Q99715 | - | ++ | Collagen alpha-1(XII) chain |
| + |  |  |  | + | + | + | P39060 | ++ | ++ | Collagen alpha-1(XVIII) chain |
| + |  |  |  | + | + | + | P08123 | ++ | +++ | Collagen alpha-2(I) chain |
| + |  |  |  | + | + | + | P12110 | ++ | ++++ | Collagen alpha-2(VI) chain |
| + |  |  | + | + | + | + | P12111 | ++++ | ++++ | Collagen alpha-3(VI) chain |
| + |  |  |  |  | + | + | A6NMZ7 | ++ | - | Collagen alpha-6(VI) chain |
| + |  |  |  | + | + | + | Q96CG8 | ++ | ++ | Collagen triple helix repeat-containing protein 1 |
| + | + |  | + |  |  |  | P78357 | ++ | - | Contactin-associated protein 1 |
|  | + |  | + |  |  |  | Q9UPY5 | ++ | - | Cystine/glutamate transporter |
|  | + |  | + |  |  |  | Q07065 | +++ | ++++ | Cytoskeleton-associated protein 4 |
|  | + |  | + |  |  |  | Q92629 | ++++ | - | Delta-sarcoglycan |
| + |  |  |  |  | + |  | P81605 | ++ | ++ | Dermcidin |
| + | + |  | + |  |  |  | Q02413 | ++ | - | Desmoglein-1 |
| + | + |  | + |  |  |  | Q96PD2 | +++ | - | Discoidin, CUB and LCCL domain-containing protein 2 |
| + | + |  | + | + | + |  | Q13443 | ++++ | ++ | Disintegrin and metalloproteinase domain-containing protein 9 |
| + |  |  |  |  | + |  | Q8IXB1 | ++ | ++ | DnaJ homolog subfamily C member 10 |
| + | + |  | + | + | + | + | Q14118 | ++++ | - | Dystroglycan |
| + |  |  |  |  | + |  | O43854 | ++ | - | EGF-like repeat and discoidin I-like domain-containing protein 3 |
|  | + |  | + |  |  |  | Q9Y6R1 | +++ | - | Electrogenic sodium bicarbonate cotransporter 1 |
| + |  |  |  |  | + | + | Q9Y6C2 | + | +++ | EMILIN-1 |
| + |  |  |  |  | + |  | O94919 | ++ | - | Endonuclease domain-containing 1 protein |
|  | + |  | + |  |  |  | P42892 | +++ | - | Endothelin-converting enzyme 1 |
| + | + |  | + |  |  |  | P29317 | ++++ | +++ | Ephrin type-A receptor 2 |
| + | + |  | + |  |  |  | P54764 | +++ | - | Ephrin type-A receptor 4 |
| + | + |  | + |  |  |  | P54756 | ++ | - | Ephrin type-A receptor 5 |
| + | + |  | + |  |  |  | P29323 | +++ | - | Ephrin type-B receptor 2 |
| + | + |  | + |  |  |  | P54753 | ++ | - | Ephrin type-B receptor 3 |
| + | + |  | + |  |  |  | P54760 | ++++ | - | Ephrin type-B receptor 4 |
| + | + |  | + |  | + |  | O15197 | +++ | - | Ephrin type-B receptor 6 |
| + |  | + | + |  |  |  | P52803 | ++ | - | Ephrin-A5 |
| + | + |  | + |  |  |  | P98172 | +++ | - | Ephrin-B1 |
| + | + |  | + |  |  |  | P52799 | ++ | - | Ephrin-B2 |
| + | + |  | + | + | + |  | P00533 | ++++ | +++ | Epidermal growth factor receptor |
|  | + |  | + |  |  |  | O43556 | +++ | - | Epsilon-sarcoglycan |
|  | + |  | + |  |  |  | Q99808 | ++ | - | Equilibrative nucleoside transporter 1 |
|  | + |  | + |  |  |  | O94905 | +++ | +++ | Erlin-2 |
| + |  |  |  |  | + |  | Q8NBQ5 | ++ | ++ | Estradiol 17-beta-dehydrogenase 11 |
|  | + |  | + |  |  |  | P43005 | ++ | - | Excitatory amino acid transporter 3 |
|  | + |  | + |  |  |  | A0FGR8 | +++ | +++ | Extended synaptotagmin-2 |
|  | + |  | + |  |  |  | O95864 | +++ | +++ | Fatty acid desaturase 2 |
|  | + |  | + |  |  |  | Q9UPI3 | ++ | - | Feline leukemia virus subgroup C receptor-related protein 2 |
| + |  |  |  | + | + | + | P02751 | +++ | ++++ | Fibronectin |
| + |  |  |  | + | + | + | P23142 | ++ | - | Fibulin-1 |
|  | + |  | + |  |  |  | P41440 | ++ | - | Folate transporter 1 |
| + | + |  | + |  |  |  | Q9UP38 | ++ | - | Frizzled-1 |
| + | + |  | + |  |  |  | Q14332 | +++ | - | Frizzled-2 |
| + | + |  | + |  |  |  | O75084 | +++ | - | Frizzled-7 |
| + |  |  |  | + | + | + | Q08380 | ++ | - | Galectin-3-binding protein |
| + |  |  |  | + | + |  | Q92820 | ++ | ++ | Gamma-glutamyl hydrolase |
|  | + |  | + |  |  |  | P36269 | ++ | - | Gamma-glutamyltransferase 5 |
|  | + |  | + |  |  |  | P17302 | ++++ | +++ | Gap junction alpha-1 protein |
| + |  | + | + |  |  |  | P56159 | ++ | - | GDNF family receptor alpha-1 |
| + |  |  |  | + | + |  | P06396 | - | ++++ | Gelsolin |
| + |  |  | + | + | + | + | P07093 | + | - | Glia-derived nexin |
| + | + |  | + |  | + |  | P48060 | +++ | - | Glioma pathogenesis-related protein 1 |
| + |  |  |  |  | + |  | Q96SL4 | ++ | - | Glutathione peroxidase 7 |
| + |  | + | + | + | + | + | P35052 | ++++ | +++ | Glypican-1 |
| + |  | + | + | + | + | + | Q9Y625 | ++ | - | Glypican-6 |
| + | + |  |  |  |  | + | Q92896 | ++++ | +++ | Golgi apparatus protein 1 |
| + | + |  | + |  |  |  | Q96PE1 | +++ | - | G-protein coupled receptor 124 |
| + |  |  |  | + | + |  | O60565 | ++ | +++ | Gremlin-1 |
| + |  | + | + |  |  |  | P54826 | ++ | - | Growth arrest-specific protein 1 |
| + |  |  |  | + | + |  | Q14393 | +++ | - | Growth arrest-specific protein 6 |
| + |  |  |  | + | + |  | P00738 | ++ | - | Haptoglobin |
| + |  |  |  | + | + |  | P02790 | ++ | - | Hemopexin |
| + | + |  | + |  |  |  | Q30201 | ++ | - | Hereditary hemochromatosis protein |
|  | + |  | + |  |  |  | P30825 | +++ | - | High affinity cationic amino acid transporter 1 |
| + | + |  | + |  |  |  | P30443 | ++++ | - | HLA class I histocompatibility antigen, A-1 alpha chain |
| + | + |  | + |  |  |  | P01892 | ++++ | - | HLA class I histocompatibility antigen, A-2 alpha chain |
| + | + |  | + |  |  |  | P05534 | ++ | - | HLA class I histocompatibility antigen, A-24 alpha chain |
| + | + |  | + |  |  |  | P04439 | - | +++ | HLA class I histocompatibility antigen, A-3 alpha chain |
| + | + |  | + |  |  |  | P30481 | ++ | - | HLA class I histocompatibility antigen, B-44 alpha chain |
| + | + |  | + |  |  |  | P30460 | ++++ | - | HLA class I histocompatibility antigen, B-8 alpha chain |
| + | + |  | + |  |  |  | P30504 | +++ | - | HLA class I histocompatibility antigen, Cw-4 alpha chain |
| + | + |  | + |  |  |  | P10321 | +++ | - | HLA class I histocompatibility antigen, Cw-7 alpha chain |
| + |  | + | + |  |  |  | Q12891 | ++ | - | Hyaluronidase-2 |
| + | + |  | + |  |  |  | P55899 | +++ | - | IgG receptor FcRn large subunit p51 |
| + | + |  | + |  |  |  | Q8TDY8 | +++ | - | Immunoglobulin superfamily DCC subclass member 4 |
|  | + |  | + |  |  |  | Q14573 | ++ | - | Inositol 1,4,5-trisphosphate receptor type 3 |
|  | + |  | + |  |  |  | Q9NQX7 | ++ | - | Integral membrane protein 2C |
| + | + |  | + |  |  |  | O75578 | +++ | ++ | Integrin alpha-10 |
| + | + |  | + |  |  |  | Q13683 | +++ | - | Integrin alpha-7 |
| + | + |  | + |  |  |  | P53708 | +++ | +++ | Integrin alpha-8 |
| + | + |  | + |  |  |  | P18084 | ++++ | - | Integrin beta-5 |
| + | + |  | + |  |  |  | Q9UMF0 | + | - | Intercellular adhesion molecule 5 |
|  | + |  | + |  |  |  | Q01628 | +++ | - | Interferon-induced transmembrane protein 3 |
| + | + |  | + |  | + |  | Q9NPH3 | ++ | - | Interleukin-1 receptor accessory protein |
| + | + |  | + | + | + |  | Q9BX67 | +++ | - | Junctional adhesion molecule C |
| + | + |  | + |  |  |  | Q96J84 | +++ | - | Kin of IRRE-like protein 1 |
|  | + |  | + |  |  |  | Q86UP2 | ++ | +++ | Kinectin |
| + |  |  | + | + | + |  | Q08431 | +++ | ++ | Lactadherin |
| + |  |  |  | + | + | + | P07942 | - | +++ | Laminin subunit beta-1 |
| + |  |  |  |  | + | + | P55268 | - | ++ | Laminin subunit beta-2 |
| + |  |  |  | + | + | + | P11047 | - | +++ | Laminin subunit gamma-1 |
|  | + |  | + |  |  |  | Q01650 | ++++ | - | Large neutral amino acids transporter small subunit 1 |
| + | + |  | + |  |  |  | O95490 | +++ | - | Latrophilin-2 |
| + | + |  | + |  | + | + | O43155 | ++ | - | Leucine-rich repeat transmembrane protein FLRT2 |
| + | + |  | + |  | + | + | Q9NZU0 | +++ | - | Leucine-rich repeat transmembrane protein FLRT3 |
|  | + |  | + |  | + |  | Q9UIQ6 | +++ | ++ | Leucyl-cystinyl aminopeptidase |
| + |  | + | + |  |  |  | Q13449 | ++ | - | Limbic system-associated membrane protein |
| + | + |  | + |  |  |  | Q86UK5 | ++ | - | Limbin |
|  | + |  | + |  |  |  | O14495 | +++ | ++ | Lipid phosphate phosphohydrolase 3 |
|  | + |  | + |  |  |  | P52569 | ++ | - | Low affinity cationic amino acid transporter 2 |
| + | + |  | + | + | + |  | P01130 | +++ | ++ | Low-density lipoprotein receptor |
| + | + |  | + |  |  |  | O75581 | ++ | - | Low-density lipoprotein receptor-related protein 6 |
|  | + |  | + |  |  |  | Q92633 | ++++ | - | Lysophosphatidic acid receptor 1 |
|  | + |  | + |  |  |  | Q14108 | ++ | +++ | Lysosome membrane protein 2 |
| + |  |  |  | + | + |  | P61626 | ++ | - | Lysozyme C |
| + | + |  | + | + | + |  | P09603 | ++ | - | Macrophage colony-stimulating factor 1 |
| + | + |  | + |  |  |  | Q9H0U3 | ++ | - | Magnesium transporter protein 1 |
| + |  |  |  |  | + |  | Q9UM22 | +++ | - | Mammalian ependymin-related protein 1 |
|  | + |  | + |  |  |  | Q96AM1 | +++ | - | Mas-related G-protein coupled receptor member F |
| + | + |  | + |  |  | + | P50281 | ++++ | +++ | Matrix metalloproteinase-14 |
| + |  |  |  |  | + |  | Q9NR99 | ++ | ++ | Matrix-remodeling-associated protein 5 |
|  | + |  | + |  |  |  | Q5HYA8 | ++ | - | Meckelin |
| + |  |  |  | + | + |  | P55145 | - | ++ | Mesencephalic astrocyte-derived neurotrophic factor |
|  | + |  | + |  |  |  | Q8NE01 | ++ | - | Metal transporter CNNM3 |
|  | + |  | + |  |  |  | Q6P4Q7 | ++ | - | Metal transporter CNNM4 |
| + |  |  |  |  | + | + | P01033 | ++ | - | Metalloproteinase inhibitor 1 |
|  | + |  | + |  |  |  | Q9UHE8 | +++ | - | Metalloreductase STEAP1 |
|  | + |  | + |  |  |  | Q8NFT2 | ++ | - | Metalloreductase STEAP2 |
|  | + |  | + |  |  |  | Q658P3 | +++ | - | Metalloreductase STEAP3 |
| + | + |  | + | + | + |  | Q29983 | ++ | - | MHC class I polypeptide-related sequence A |
|  | + |  | + |  |  |  | Q8TCT9 | +++ | +++ | Minor histocompatibility antigen H13 |
|  | + |  | + |  |  |  | Q02978 | +++ | +++ | Mitochondrial 2-oxoglutarate/malate carrier protein |
|  | + |  | + |  |  |  | Q8IXI2 | ++ | - | Mitochondrial Rho GTPase 1 |
|  | + |  | + |  |  |  | Q8IXI1 | ++ | - | Mitochondrial Rho GTPase 2 |
|  | + |  | + |  |  |  | P53985 | ++++ | ++ | Monocarboxylate transporter 1 |
|  | + |  | + |  |  |  | O60669 | ++ | - | Monocarboxylate transporter 2 |
|  | + |  | + |  |  |  | O15427 | ++++ | +++ | Monocarboxylate transporter 4 |
|  | + |  | + |  |  |  | O15374 | ++ | - | Monocarboxylate transporter 5 |
|  | + |  | + |  |  |  | P36021 | +++ | - | Monocarboxylate transporter 8 |
|  | + |  | + |  |  |  | P33527 | ++++ | - | Multidrug resistance-associated protein 1 |
|  | + |  | + |  |  |  | O15439 | +++ | - | Multidrug resistance-associated protein 4 |
| + | + |  | + |  |  |  | O95297 | ++++ | - | Myelin protein zero-like protein 1 |
|  | + |  | + |  |  |  | Q9NZM1 | ++++ | ++++ | Myoferlin |
| + |  |  |  |  | + |  | Q96PD5 | ++ | - | N-acetylmuramoyl-L-alanine amidase |
|  | + |  | + |  |  |  | Q9Y2A7 | ++ | +++ | Nck-associated protein 1 |
| + | + |  | + |  |  |  | Q92859 | ++++ | ++ | Neogenin |
| + |  | + | + |  |  |  | Q9Y2I2 | ++ | - | Netrin-G1 |
| + | + |  | + |  |  |  | O15394 | ++ | - | Neural cell adhesion molecule 2 |
| + | + |  | + |  | + | + | O00533 | ++ | - | Neural cell adhesion molecule L1-like protein |
| + | + |  | + |  |  |  | O94856 | ++++ | - | Neurofascin |
| + | + |  | + |  | + |  | P46531 | ++ | - | Neurogenic locus notch homolog protein 1 |
| + | + |  | + |  | + |  | Q04721 | ++++ | ++ | Neurogenic locus notch homolog protein 2 |
| + | + |  | + |  | + |  | Q9UM47 | +++ | - | Neurogenic locus notch homolog protein 3 |
| + | + |  | + |  |  |  | Q8N2Q7 | ++ | - | Neuroligin-1 |
| + | + |  | + |  |  |  | Q8NFZ4 | ++ | - | Neuroligin-2 |
| + | + |  | + |  |  |  | Q92823 | ++ | - | Neuronal cell adhesion molecule |
| + |  | + | + |  |  |  | Q7Z3B1 | +++ | - | Neuronal growth regulator 1 |
|  | + |  | + |  |  |  | Q13491 | ++ | - | Neuronal membrane glycoprotein M6-b |
| + | + |  | + |  |  |  | O60462 | ++ | - | Neuropilin-2 |
| + | + |  | + |  |  |  | Q9Y639 | ++++ | +++ | Neuroplastin |
| + |  | + | + |  |  |  | Q9P121 | +++ | - | Neurotrimin |
|  | + |  | + |  |  |  | P43007 | +++ | - | Neutral amino acid transporter A |
|  | + |  | + |  |  |  | Q15758 | ++++ | +++ | Neutral amino acid transporter B(0) |
| + |  |  |  | + | + |  | P59665 | + | - | Neutrophil defensin 1 |
| + | + |  | + |  |  |  | Q92542 | ++++ | +++ | Nicastrin |
| + |  |  |  |  | + | + | Q14112 | ++ | - | Nidogen-2 |
| + |  | + | + |  |  |  | Q9BZM4 | ++ | - | NKG2D ligand 3 |
| + | + |  | + |  |  |  | Q99650 | +++ | - | Oncostatin-M-specific receptor subunit beta |
|  | + |  | + |  |  |  | Q96RD7 | +++ | - | Pannexin-1 |
| + |  |  |  |  | + |  | P26022 | + | - | Pentraxin-related protein PTX3 |
| + |  |  |  | + | + | + | Q92626 | ++ | +++ | Peroxidasin homolog |
| + |  |  |  | + | + |  | Q13162 | +++ | +++ | Peroxiredoxin-4 |
|  | + |  | + |  |  |  | Q00325 | ++++ | ++++ | Phosphate carrier protein, mitochondrial |
|  | + |  | + |  |  | + | O15162 | +++ | - | Phospholipid scramblase 1 |
|  | + |  | + |  |  |  | Q9NRY6 | - | +++ | Phospholipid scramblase 3 |
|  | + |  | + |  |  |  | P20020 | ++++ | ++ | Plasma membrane calcium-transporting ATPase 1 |
|  | + |  | + |  |  |  | P20020 | ++++ | ++ | Plasma membrane calcium-transporting ATPase 1 |
|  | + |  | + |  |  |  | P23634 | ++++ | ++++ | Plasma membrane calcium-transporting ATPase 4 |
| + |  |  | + | + | + | + | P05121 | +++ | +++ | Plasminogen activator inhibitor 1 |
| + | + |  | + |  |  |  | Q5VY43 | +++ | - | Platelet endothelial aggregation receptor 1 |
| + | + |  | + |  |  |  | Q9UIW2 | ++++ | - | Plexin-A1 |
| + | + |  | + |  |  |  | O75051 | +++ | - | Plexin-A2 |
| + | + |  | + |  |  |  | P51805 | +++ | - | Plexin-A3 |
| + | + |  | + |  | + |  | O43157 | ++ | - | Plexin-B1 |
| + | + |  | + |  |  |  | O15031 | ++++ | - | Plexin-B2 |
| + | + |  | + |  |  |  | Q9Y4D7 | ++++ | - | Plexin-D1 |
|  | + |  | + |  |  |  | Q13563 | ++ | - | Polycystin-2 |
| + |  |  | + | + | + |  | Q9UHG3 | ++ | +++ | Prenylcysteine oxidase 1 |
|  | + |  | + |  |  |  | P49768 | +++ | - | Presenilin-1 |
| + |  |  |  | + | + |  | P07602 | - | +++ | Proactivator polypeptide |
|  | + |  | + |  |  |  | Q14439 | +++ | - | Probable G-protein coupled receptor 176 |
|  | + |  | + |  |  |  | Q9C0B5 | ++ | - | Probable palmitoyltransferase ZDHHC5 |
|  | + |  | + |  |  |  | Q9Y2G3 | +++ | - | Probable phospholipid-transporting ATPase IF |
|  | + |  | + |  |  |  | Q8NB49 | ++++ | - | Probable phospholipid-transporting ATPase IG |
|  | + |  | + |  |  |  | P98196 | ++ | - | Probable phospholipid-transporting ATPase IH |
|  | + |  | + |  |  |  | Q9P241 | ++ | - | Probable phospholipid-transporting ATPase VD |
| + |  |  |  | + | + |  | Q15113 | ++ | - | Procollagen C-endopeptidase enhancer 1 |
|  | + |  | + |  |  |  | Q9HCJ1 | ++ | - | Progressive ankylosis protein homolog |
| + |  |  | + |  | + |  | P12273 | ++ | - | Prolactin-inducible protein |
| + |  |  |  |  | + | + | Q32P28 | - | +++ | Prolyl 3-hydroxylase 1 |
|  | + |  | + |  |  |  | P43116 | ++ | - | Prostaglandin E2 receptor EP2 subtype |
|  | + |  | + |  |  |  | Q8TCG1 | + | - | Protein CIP2A |
| + |  |  |  |  | + |  | O00622 | + | +++ | Protein CYR61 |
| + |  |  | + |  | + |  | P07237 | +++ | ++++ | Protein disulfide-isomerase |
| + |  |  |  |  | + |  | Q92520 | ++ | ++ | Protein FAM3C |
| + | + |  | + |  | + |  | Q9ULI3 | ++ | - | Protein HEG homolog 1 |
|  | + |  | + |  |  |  | Q86UE4 | ++ | +++ | Protein LYRIC |
|  | + |  | + |  |  |  | Q92508 | +++ | ++ | Protein PIEZO1 |
|  | + |  | + |  |  |  | Q9C0H2 | +++ | ++ | Protein tweety homolog 3 |
| + |  |  | + | + | + | + | P41221 | ++ | - | Protein Wnt-5a |
| + |  |  | + | + | + | + | Q9H1J7 | ++ | - | Protein Wnt-5b |
| + | + |  | + |  | + |  | P25116 | +++ | - | Proteinase-activated receptor 1 |
| + |  |  |  | + | + | + | P28300 | ++ | - | Protein-lysine 6-oxidase |
|  | + |  | + |  |  |  | Q04941 | +++ | - | Proteolipid protein 2 |
| + | + |  | + |  |  |  | Q6V0I7 | +++ | - | Protocadherin Fat 4 |
| + | + |  | + |  |  |  | Q9Y5H3 | ++ | - | Protocadherin gamma-A10 |
| + | + |  | + |  |  |  | Q9Y5G0 | +++ | - | Protocadherin gamma-B5 |
| + | + |  | + |  |  |  | Q9Y5F8 | ++ | - | Protocadherin gamma-B7 |
| + | + |  | + |  |  |  | Q9UN70 | ++++ | - | Protocadherin gamma-C3 |
| + | + |  | + |  |  |  | Q96JQ0 | ++ | - | Protocadherin-16 |
| + | + |  | + |  |  |  | Q9HCL0 | +++ | - | Protocadherin-18 |
| + | + |  | + |  |  |  | Q9HC56 | ++ | - | Protocadherin-9 |
| + | + |  | + |  |  |  | Q68D85 | + | - | Putative Ig-like domain-containing protein |
|  | + |  | + |  |  |  | Q9BSK0 | +++ | - | Putative MARVEL domain-containing protein 1 |
| + | + |  | + |  |  |  | P18433 | +++ | - | Receptor-type tyrosine-protein phosphatase alpha |
| + | + |  | + |  |  |  | P10586 | +++ | - | Receptor-type tyrosine-protein phosphatase F |
| + | + |  | + |  |  |  | P23470 | ++++ | - | Receptor-type tyrosine-protein phosphatase gamma |
| + | + |  | + |  |  |  | Q15262 | +++ | - | Receptor-type tyrosine-protein phosphatase kappa |
| + | + |  | + |  |  |  | P28827 | +++ | - | Receptor-type tyrosine-protein phosphatase mu |
| + | + |  | + |  |  |  | Q13332 | +++ | - | Receptor-type tyrosine-protein phosphatase S |
| + | + |  | + |  |  |  | Q8IUW5 | +++ | - | RELT-like protein 1 |
| + | + |  | + |  |  |  | O75787 | ++ | - | Renin receptor |
|  | + |  | + |  |  |  | Q9NQC3 | ++++ | ++++ | Reticulon-4 |
| + |  | + | + |  |  |  | O95980 | +++ | - | Reversion-inducing cysteine-rich protein with Kazal motifs |
| + |  | + | + |  |  |  | Q6NW40 | +++ | - | RGM domain family member B |
|  | + |  | + |  |  |  | Q9HAB3 | +++ | - | Riboflavin transporter 3 |
| + | + |  | + |  |  |  | Q9Y6N7 | ++++ | - | Roundabout homolog 1 |
| + | + |  | + |  |  |  | Q9HCK4 | +++ | - | Roundabout homolog 2 |
|  | + |  | + |  |  |  | P16615 | ++++ | ++++ | Sarcoplasmic/endoplasmic reticulum calcium ATPase 2 |
| + | + |  | + | + | + |  | Q8WVN6 | ++ | - | Secreted and transmembrane protein 1 |
| + |  |  | + | + | + | + | Q8N474 | ++ | ++ | Secreted frizzled-related protein 1 |
|  | + |  | + | + | + |  | Q12884 | ++++ | +++ | Seprase |
|  | + |  | + |  |  |  | Q9NRX5 | ++ | - | Serine incorporator 1 |
| + |  |  |  | + | + |  | Q92743 | - | ++ | Serine protease HTRA1 |
| + |  |  | + |  | + |  | Q15165 | ++ | - | Serum paraoxonase/arylesterase 2 |
|  | + |  | + |  |  |  | Q99720 | +++ | ++ | Sigma non-opioid intracellular receptor 1 |
| + | + |  | + |  |  |  | Q8TCT8 | +++ | - | Signal peptide peptidase-like 2A |
| + | + |  | + |  |  |  | Q8TCT7 | ++ | - | Signal peptide peptidase-like 2B |
|  | + |  | + |  |  |  | P31641 | +++ | - | Sodium- and chloride-dependent taurine transporter |
|  | + |  | + |  |  |  | Q9Y6M7 | ++ | - | Sodium bicarbonate cotransporter 3 |
| + | + |  | + |  |  |  | P32418 | +++ | - | Sodium/calcium exchanger 1 |
|  | + |  | + |  |  |  | P19634 | +++ | - | Sodium/hydrogen exchanger 1 |
|  | + |  | + |  |  |  | Q92581 | ++ | - | Sodium/hydrogen exchanger 6 |
|  | + |  | + |  |  |  | P53794 | ++ | - | Sodium/myo-inositol cotransporter |
|  | + |  | + |  |  |  | P05023 | ++++ | ++++ | Sodium/potassium-transporting ATPase subunit alpha-1 |
|  | + |  | + |  |  |  | P05026 | ++++ | - | Sodium/potassium-transporting ATPase subunit beta-1 |
|  | + |  | + |  |  |  | Q96QD8 | +++ | +++ | Sodium-coupled neutral amino acid transporter 2 |
|  | + |  | + |  |  |  | Q8WUX1 | ++ | - | Sodium-coupled neutral amino acid transporter 5 |
|  | + |  | + |  |  |  | Q9Y289 | +++ | - | Sodium-dependent multivitamin transporter |
|  | + |  | + |  |  |  | Q8WUM9 | ++ | - | Sodium-dependent phosphate transporter 1 |
|  | + |  | + |  |  |  | Q08357 | ++ | - | Sodium-dependent phosphate transporter 2 |
|  | + |  | + |  |  |  | P55011 | + | - | Solute carrier family 12 member 2 |
|  | + |  | + |  |  |  | Q9UP95 | +++ | - | Solute carrier family 12 member 4 |
|  | + |  | + |  |  |  | Q9UHW9 | ++ | - | Solute carrier family 12 member 6 |
|  | + |  | + |  |  |  | Q9BXP2 | ++ | - | Solute carrier family 12 member 9 |
|  | + |  | + |  |  |  | P11166 | ++++ | +++ | Solute carrier family 2, facilitated glucose transporter member 1 |
|  | + |  | + |  |  |  | O95528 | ++ | - | Solute carrier family 2, facilitated glucose transporter member 10 |
|  | + |  | + |  |  |  | Q96BI1 | ++ | - | Solute carrier family 22 member 18 |
| + | + |  | + |  |  |  | Q99523 | ++ | - | Sortilin |
| + |  |  |  | + | + | + | P09486 | ++ | - | SPARC |
|  | + |  | + |  |  |  | O15121 | +++ | - | Sphingolipid delta(4)-desaturase DES1 |
|  | + |  | + |  |  |  | O95136 | ++ | - | Sphingosine 1-phosphate receptor 2 |
|  | + |  | + |  |  |  | Q99500 | ++ | - | Sphingosine 1-phosphate receptor 3 |
| + |  |  | + | + | + |  | P48061 | + | - | Stromal cell-derived factor 1 |
|  | + |  | + |  |  |  | P50443 | ++ | - | Sulfate transporter |
| + | + |  |  | + | + |  | O00391 | ++ | - | Sulfhydryl oxidase 1 |
| + | + |  | + |  | + |  | Q6ZRP7 | + | - | Sulfhydryl oxidase 2 |
|  | + |  | + |  |  |  | O43760 | ++ | - | Synaptogyrin-2 |
|  | + |  | + |  |  |  | Q16563 | ++++ | - | Synaptophysin-like protein 1 |
| + | + |  | + |  | + |  | P31431 | +++ | - | Syndecan-4 |
|  | + |  | + |  |  |  | Q12846 | +++ | - | Syntaxin-4 |
|  | + |  | + |  |  |  | Q9UNK0 | ++ | ++ | Syntaxin-8 |
| + | + |  |  |  | + |  | Q8TB96 | ++ | - | T-cell immunomodulatory protein |
| + |  |  |  | + | + | + | P24821 | - | +++ | Tenascin |
| + |  |  |  |  | + |  | Q9Y6I9 | ++ | - | Testis-expressed sequence 264 protein |
|  | + |  | + |  |  |  | O14817 | ++ | - | Tetraspanin-4 |
|  | + |  | + |  |  |  | O75954 | +++ | - | Tetraspanin-9 |
| + | + |  | + |  |  |  | P36897 | ++ | - | TGF-beta receptor type-1 |
| + | + |  | + |  |  |  | P37173 | +++ | - | TGF-beta receptor type-2 |
|  | + |  | + |  |  |  | Q9UKU6 | +++ | - | Thyrotropin-releasing hormone-degrading ectoenzyme |
| + | + |  | + | + | + | + | Q03167 | +++ | - | Transforming growth factor beta receptor type 3 |
| + |  |  | + | + | + | + | Q15582 | ++ | +++ | Transforming growth factor-beta-induced protein ig-h3 |
|  | + |  | + |  |  |  | Q8TD43 | ++ | - | Transient receptor potential cation channel subfamily M member 4 |
|  | + |  | + |  |  |  | Q9Y5S1 | +++ | ++ | Transient receptor potential cation channel subfamily V member 2 |
| + | + |  | + |  |  |  | Q99805 | - | ++ | Transmembrane 9 superfamily member 2 |
| + | + |  | + |  |  |  | P49755 | +++ | +++ | Transmembrane emp24 domain-containing protein 10 |
|  | + |  | + |  | + |  | O14668 | ++ | - | Transmembrane gamma-carboxyglutamic acid protein 1 |
| + | + |  | + |  |  |  | Q14956 | ++ | - | Transmembrane glycoprotein NMB |
|  | + |  | + |  |  |  | Q86WV6 | ++ | ++ | Transmembrane protein 173 |
|  | + |  | + |  |  |  | Q9BQJ4 | ++ | - | Transmembrane protein 47 |
|  | + |  | + |  |  |  | Q96GC9 | ++ | - | Transmembrane protein 49 |
| + | + |  | + |  |  |  | Q13641 | ++++ | ++ | Trophoblast glycoprotein |
| + | + |  | + | + | + |  | P30530 | +++ | ++ | Tyrosine-protein kinase receptor UFO |
| + | + |  | + |  |  |  | Q01973 | ++ | - | Tyrosine-protein kinase transmembrane receptor ROR1 |
| + | + |  | + |  |  |  | Q13308 | ++++ | +++ | Tyrosine-protein kinase-like 7 |
| + |  |  |  |  | + |  | Q969H8 | - | +++ | UPF0556 protein C19orf10 |
| + | + |  |  |  | + |  | Q6EMK4 | ++++ | +++ | Vasorin |
|  | + |  | + |  |  |  | P63027 | +++ | - | Vesicle-associated membrane protein 2 |
|  | + |  | + |  |  |  | Q15836 | - | +++ | Vesicle-associated membrane protein 3 |
|  | + |  | + |  |  |  | O95183 | ++ | - | Vesicle-associated membrane protein 5 |
|  | + |  | + |  |  |  | Q9P0L0 | +++ | +++ | Vesicle-associated membrane protein-associated protein A |
|  | + |  | + |  |  |  | P21796 | +++ | ++++ | Voltage-dependent anion-selective channel protein 1 |
| + | + |  | + |  |  |  | P54289 | ++++ | +++ | Voltage-dependent calcium channel subunit alpha-2/delta-1 |
|  | + |  | + |  |  |  | Q93050 | ++ | - | V-type proton ATPase 116 kDa subunit a isoform 1 |
|  | + |  | + |  |  |  | Q13488 | ++ | +++ | V-type proton ATPase 116 kDa subunit a isoform 3 |
|  | + |  | + |  |  |  | Q92536 | +++ | - | Y+L amino acid transporter 2 |
|  | + |  | + |  |  |  | Q9Y6M5 | +++ | - | Zinc transporter 1 |
| + | + |  | + |  |  |  | Q15043 | +++ | - | Zinc transporter ZIP14 |
| + | + |  | + |  |  |  | Q13433 | ++ | - | Zinc transporter ZIP6 |
| + |  |  | + |  | + |  | P25311 | + | - | Zinc-alpha-2-glycoprotein |

Identified proteins were accepted as cell surface proteins if they are GPI-anchored (GA) or transmembrane proteins (TM) assigned to the plasma membrane (PM) or signal peptide-containing proteins (SP) that are assigned to either the extracellular space (ECS), region (ECR) or matrix (ECM).
